# Adaptive information processing of network modules to dynamic and spatial stimuli

**DOI:** 10.1186/s12918-019-0703-1

**Published:** 2019-03-14

**Authors:** J. Krishnan, Ioannis Floros

**Affiliations:** 1 Department of Chemical Engineering, Centre for Process Systems Engineering, Imperial College London, South Kensington, London, SW7 2AZ UK; 2 National Centre of Scientific Research “Demokritos”, Athens, Greece

**Keywords:** Adaptation, Dynamic stimuli, Spatial stimuli, Network features

## Abstract

**Background:**

Adaptation and homeostasis are basic features of information processing in cells and seen in a broad range of contexts. Much of the current understanding of adaptation in network modules/motifs is based on their response to simple stimuli. Recently, there have also been studies of adaptation in dynamic stimuli. However a broader synthesis of how different circuits of adaptation function, and which circuits enable a broader adaptive behaviour in classes of more complex and spatial stimuli is largely missing.

**Results:**

We study the response of a variety of adaptive circuits to time-varying stimuli such as ramps, periodic stimuli and static and dynamic spatial stimuli. We find that a variety of responses can be seen in ramp stimuli, making this a basis for discriminating between even similar circuits. We also find that a number of circuits adapt exactly to ramp stimuli, and dissect these circuits to pinpoint what characteristics (architecture, feedback, biochemical aspects, information processing ingredients) allow for this. These circuits include incoherent feedforward motifs, inflow-outflow motifs and transcritical circuits. We find that changes in location in such circuits where a signal acts can result in non-adaptive behaviour in ramps, even though the location was associated with exact adaptation in step stimuli. We also demonstrate that certain augmentations of basic inflow-outflow motifs can alter the behaviour of the circuit from exact adaptation to non-adaptive behaviour. When subject to periodic stimuli, some circuits (inflow-outflow motifs and transcritical circuits) are able to maintain an average output independent of the characteristics of the input. We build on this to examine the response of adaptive circuits to static and dynamic spatial stimuli. We demonstrate how certain circuits can exhibit a graded response in spatial static stimuli with an exact maintenance of the spatial mean-value. Distinct features which emerge from the consideration of dynamic spatial stimuli are also discussed. Finally, we also build on these results to show how different circuits which show any combination of presence or absence of exact adaptation in ramps, exact mainenance of time average output in periodic stimuli and exact maintenance of spatial average of output in static spatial stimuli may be realized.

**Conclusions:**

By studying a range of network circuits/motifs on one hand and a range of stimuli on the other, we isolate characteristics of these circuits (structural) which enable different degrees of exact adaptive and homeostatic behaviour in such stimuli, how they may be combined, and also identify cases associated with non-homeostatic behaviour. We also reveal constraints associated with locations where signals may act to enable homeostatic behaviour and constraints associated with augmentations of circuits. This consideration of multiple experimentally/naturally relevant stimuli along with circuits of adaptation of relevance in natural and engineered biology, provides a platform for deepening our understanding of adaptive and homeostatic behaviour in natural systems, bridging the gap between models of adaptation and experiments and in engineering homeostatic synthetic circuits.

**Electronic supplementary material:**

The online version of this article (10.1186/s12918-019-0703-1) contains supplementary material, which is available to authorized users.

## Background

Cellular systems employ a number of distinct and characteristic nonlinear information processing modules, such as monostable switches, bistable switches and oscillators. Each of these modules plays critical roles in cells, and consequently such modules has been a focal point in a number of cellular contexts [1–5]. A particular information processing characteristic repeatedly encountered in cellular networks is adapation. Adaptation is the characteristic of a module wherein the output of the module is essentially independent of the input at steady state, even though the input is “connected" to the output. A confluence of different characteristics of the module allows for this special form of information processing.

Adaptation in cellular networks is seen in multiple contexts and with different consequences. One common context in which adaptation is seen is in sensory transduction. In the context of chemotaxis (directed cellular migration in response to gradients of chemical concentrations) adaptation is observed in a range of cell types including bacteria (E.coli, rhodobacter spheroides) and eukaryotes (Dictyostelium) [6–10]. The fact that adaptation is present right at the sensory level allows bacteria to exhibit sensitivity to temporal gradients over a very broad range of ambient mean concentrations. In the case of Dictyostelium, adaptation to spatially uniform stimuli is seen alongside non-adaptive behaviour in spatial gradients. In both these cases, it appears that adaptation has been incorporated, through evolution, into signal transduction to realize specific capabilities for cells. Another context in which adaptation is seen playing a similar role, is in visual signal transduction [11–13]. Adaptation is also seen in other signal transduction settings such as osmoregulation, studied for instance in yeast, and in the heat shock response [14–17]. Finally, homeostasis in cellular systems in response to different changes in the environment, is associated with adaptive behaviour of this kind, an example being iron homeostasis in bacteria [18–24].

A fairly broad range of studies have focussed on different aspects of adaptation in biochemical and genetic networks. On one hand there are a number of experimental studies of adaptation in specific contexts, including those listed above. These studies show how adaptation occurs in the relevant circuits/pathways and what the cellular implications are. On the theoretical side, in addition to modelling the adaptive modules in various contexts, studies have focussed on a number of related information processing aspects. Exact adaptation and in particular robust exact adaptation has been widely studied, discriminating it from non-robust adaptation and focussing on the integral control underpinnings (eg. [23, 25–29]). Motifs which have resulted in adaptation have been studied widely. For instance [30] studied a range of motifs involving inflow and outflow resulting in exact adaptation. This was further expanded to study inflow and outflow controllers in adaptation [31]. An exhaustive computational study of 3-node motifs revealed incoherent feedforward and negative feedback as the two adaptive motifs which emerge [32]. Studies of information processing in adaptive motifs have been performed in [30, 33–40] focussing on spatial and temporal behaviour of incoherent adaptive circuits, response of motifs to oscillatory stimuli, spatial and stochastic aspects of adaptive signal transduction. Fold-adaptation which incorporates adaptation with a fixed fold change behaviour has also been the focus of numerous studies [9, 41–43]. Finally adaptive behaviour has also been engineered in genetic circuits in synthetic biology (eg. see [44]).

While there have been a large number of studies on adaptation, there are relatively few which study the response of adaptive modules/circuits to dynamic and complex stimuli [34, 35, 37–39]. Understanding the response of adaptive circuits and obtaining a synthesis of adaptive responses in dynamic and complex stimuli is important for multiple reasons. Firstly, this deepens our understanding of adaptive modules/circuits and shines a light on how they (and cells using these circuits) process dynamic information. Secondly a number of adaptive modules behave in a more or less similiar way to simple stimuli such as step inputs, and it is not clear at the outset whether and under which conditions such similar behaviour extends to complex dynamic stimuli. Thirdly certain dynamic and spatial stimuli have already been used in experiments, in certain contexts [45–49]. However, there a number of contexts where this aspect has not been studied, but whose deployment could provide valuable insights. Such a study has relevance in both cases. Fourthly, cellular systems are faced with dynamic and complex stimuli and dynamic environments as a norm and it is important to assess how adaptation impacts behaviour and decision-making in these environments. Since dynamic environments are the norm rather than the exception, this can provide important clues into what types of adaptive modules have emerged in evolution. Finally a broader view of adaptive circuits and their response to dynamic and spatial stimuli, suggests engineering design principles associated with circuits responding adaptively to one or more classes of complex stimuli. This serves as a basis for engineering biomolecular circuits in cells with specific adaptive and homeostatic capabilities.

In this paper, we examine the response of adaptive circuits to different transient signals such as ramp stimuli (of different types) and periodic stimuli, and subsequently spatial signals. In order to do this, we draw on a range of adaptive circuits/motifs in the literature. We investigate the response of these various modules and relate this to characteristics (structural, biochemical, auxilliary) of the circuit. Of particular interest here is the subset of adaptive circuits which exhibit degrees of exact adaptation to one or more classes of complex stimuli, and our aim is to distill the underlying characteristics responsible for this. This allows us to achieve a clear synthesis of how different circuit characteristics impact the dynamic response and enable broader adaptive behaviour.

In the next section we discuss the circuits and motifs which are employed in our study. In the subsequent section, computational results are presented. This is followed by a concise analytical discussion in the next section (with further details in the Appendix). This analytical section can be skipped, without any loss of continuity, by readers not interested in the details. The conclusions synthesize the various insights. The Additional file 1 contains further information.

## Methods

### Models

At the outset we emphasize that our goal is to obtain a synthesis of the functioning of adaptive circuits to dynamic/spatial stimuli in a systematic manner, with a particular view to determining when broader exact adaptive behaviour is seen and tracing this to circuit characteristics. Adaptation can be realized through both gene regulatory and biochemical circuits, and there are a range of models which have been used to model adaptation. We use a suite of models drawn from the literature as a basis for probing the response of adaptive circuits to dynamic environments. These models are presented in the Additional file 1. The models encompass different biological types (genetic, biochemical), different model network structures and particular characteristics. Engineering homeostatic circuits can also be realized through DNA strand displacement reactions, modelled as reaction networks, but for the most part we do not study these circuits separately.

For purposes of organization, the models are placed in a table according to two characteristics of the reaction network/motif (see Table 1). On one axis, the classification is based on how the signal appears in the model (zeroth order reaction/source), first order irreversible reaction or first order reversible reaction. Transcriptional models are classified along with the zeroth order reaction. On the second axis, the dominant characteristic responsible for adaptation in static signals is the basis for classification. The categories employed here are incoherent feedforward,negative feedback, open systems and other special characteristics. Most adaptive circuits studied fall into one of these categories. For instance incoherent feedforward and negative feedback motifs have been the focus of numerous studies in both signalling and gene regulation. Various studies including [30, 31] directly employ the fact that the network is an open system as the primary basis for adaptation. In this context, we point out that closed biochemical networks (i.e. without inflow/outlow) could result in models analogous to inflow/outflow motifs: this occurs if the only source of flux to a subnetwork from the rest of the network occurs via a zero-order reaction, while flux from the subnetwork to the rest of the network can occur through first order reactions. In such cases the subnetwork has a model essentially analogous to an inflow-outflow motif of the kind we study, with the only difference that there is coupling to the ambient network through conservation of species (this still allows for adaptive behaviour). This is especially pertinent because some negative feedback circuits (in E.coli chemotaxis) achieve exact adaptation precisely because of the presence of zeroth order reactions and the resulting model could belong to both the negative feedback and the inflow-outflow category. In this case, we briefly study it as a particular example of negative feedback (as it is the core part of a negative feedback adaptive mechanism), but draw parallels to inflow-outflow motifs. An example of a distinct behaviour responsible for adaptation is an autocatalytic circuit exhibiting a transcritical bifurcation [50, 51]. Here the system is a closed system and the feedback is a positive feedback from substrate to enzyme. This circuit has entities which reach a steady state independent of the signal. From the vantage point of the adapting variable, the governing mechanism is that of an autocatalytic negative feedback. This is treated separately as it involves a distinct ingredient responsible for adaptation, and in fact the core biochemical circuit is an example of one which has been studied in the context of absolute concentration robustness [51].

**Table 1:** List of primary models analyzed

Signal → model (reaction network) characteristic	First order reversible reaction	First order irreversible reaction	Zeroth order reaction
Feedback	MA09.FB, DR08.M4	DR08.M1**, (DR08.M6)	DR12.M1-8
Incoherent Feedforward	MA09.FF, KR11	(DR08.M7)	KR09, CO09.M1, CO09.M2, KI14
Open System	DR08.M1, DR08.M31, DR08.M32, DR08.M33, DR08.M34 (DR08.M4)	DR08.M1*, DR08.M1**, DR08.M2, DR08.M32, Dr08.M34, DR08.M6, DR08.M7	DR12.M1-8
Special Cases	TC		

We have analyzed the entire suite of models in detail. For purposes of clarity, we will present results on a smaller selection of models, which encompasses the different underpinning characteristics and the range of behaviour observed. We also examine other variants of models possessing the same characteristics responsible for adaptation, in the supplementary information. The models employed are all ODE based models (except in cases where a spatial analogue of an ODE model is considered). Further discussion of all models, along with model equations, parameters and inputs, is presented in the Additional file 1.

Model parameters are chosen according to the original model description in the literature. A number of models exhibit exact adaptation in response to a step input (i.e. exact recovery of output to prestimulus value) while a few exhibit inexact adaptation (partial recovery of output to prestimulus value). We keep parameters fixed in our study. We note here that a separation in time scale between input variation and the circuit time scale, can result in adaptive behaviour being essentially maintained (discussed later): we do not assume such a special case, and generally the time scale of input variation and adaptation are comparable.

### Inputs

In our simulations we start with a steady basal level of input, wait for the system to reach a steady state. Following this, we subject the models to different kinds of experimentally relevant inputs (a) A linear ramp (and by way of constrast also examine other increasing stimuli, such as quadratic ramps and exponential stimuli).Since a stimulus is always bounded (due to finite number of receptors or other factors), we also briefly examine ramps which saturate. (b) Periodic sinusoidal oscillations. (c) Spatially varying stimuli, including static spatial gradients, and dynamic spatiotemporal stimuli such as travelling waves and standing waves. For the purposes of presentation of responses to spatial stimuli, we focus on a smaller subset of circuits, where clear correspondences with temporal behaviour can be made (discussed later).

At the outset we note that the circuits chosen can exhibit different types of responses, depending on the nature of the stimulus. This can include (i) Exact adaptation (ii) Exact adaptation of certain features of the output (eg. mean values in response to periodic stimuli) (iii) Inexact adaptation of response or mean-values as appropriate (iv) Non-adaptive responses

Most circuits exhibit some degree of (inexact) adaptive behaviour to the stimuli considered. Our particular focus is on key qualitative features of the landscape of circuit responses, especially on circuits exhibiting different degrees of exactly adaptive behaviour in complex stimuli, where the behaviour can be traced to structural features of the model, independent of model parameters. This reveals core design features responsible for the adaptive behaviour. We also discuss non-adaptive responses, as they represent a fundamentally opposite qualitative response. We study the effect of numerically varying characteristics of inputs (or model parameters) if this is especially relevant. Our analysis and presentation of the results involves a combination of numerical simulations and mathematical analysis: numerical simulations reveals a range of different and noteworthy behaviour, while mathematical analysis reveals how certain motifs/circuits display different kinds of exact adaptive behaviour in complex stimuli. Simulations are performed in MATLAB using ode15s.

## Results

**Fig. 1:**
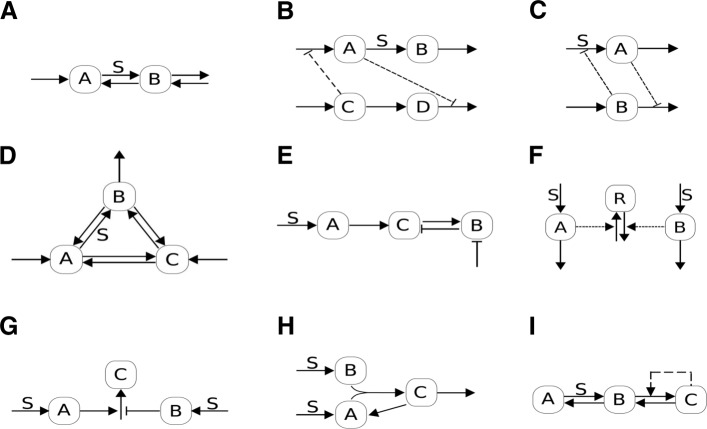
Schematic of circuits. A schematic representation of the primary motifs/circuits in the literature, employed in this paper: (**a**) Linear network structure, with inflow and outflow: model DR08.M1 (**b**, **c**) Two variants of circuits comprising two linear inflow/outflow motifs interacting with one another (DR08.M1**,DR12.M4). (**d**) A cyclic motif with inflow and outflow (DR08.M33). (**e**) A negative feedback motif (MA09.FB). (**f**, **g**, **h**) Three variants of incoherent feedforward motifs (KR09,CO09.M2,KI14) (**i**) A transcritical motif (TC). Additional variants of linear and cyclic motifs with inflow and outflow are presented in the Additional file 1: Figure S1

We have analyzed the set of models in Table 1. This reveals both similarities between different models in a given group, as well as differences, which can arise from subtle differences in details; this is also useful for determining what combinations of characteristics can exhibit exact adaptive behaviour in complex stimuli. We now comment on how we present the results. We present the results for a selection of circuits (Fig. 1), which essentially cover both the different model types, as well as distinct qualitative behaviour which we wish to demonstrate. The circuits include incoherent feedforward, feedback motifs, open systems as well as a circuit of autocatalytic feedback giving rise to a transcritical bifurcation. We employ a typical feedback motif (a 3-node motif with a buffer node) contrasting it with other feedback circuits (discussed later) and consider multiple variants of incoherent feedforward motifs. For instance models KR09 and KR11 are models of an incoherent feedforward adaptive motif developed to explain adaptation in Dictyostelium, and an expansion of that model to incorporate saturation (here and below, we refer to models through labels which are used to denote them in Additional file 1). Model CO09.M2 depicts an incoherent feedforward structure with thresholds and saturation. The model KI14, is another incoherent feedforward model exhibiting (parameter dependent approximate) fold adaptation. Similarly multiple variants of open circuits are possible. Figure 1 shows both linear and cyclic motifs DR08.M1 and DR08.M33 (which both exhibit perfect adaptation in a step stimulus), while other models involving open systems involve regulation of either inflow or outflow: a representative candidate is the model Dr12.M4 shown in Fig. 1. We present a selection of computational results to reveal the range of behaviour. This is followed by analytical results which focusses on explaining when adaptation in dynamic stimuli may be observed and what the associated design principles are.

### Ramp stimuli

**Fig. 2:**
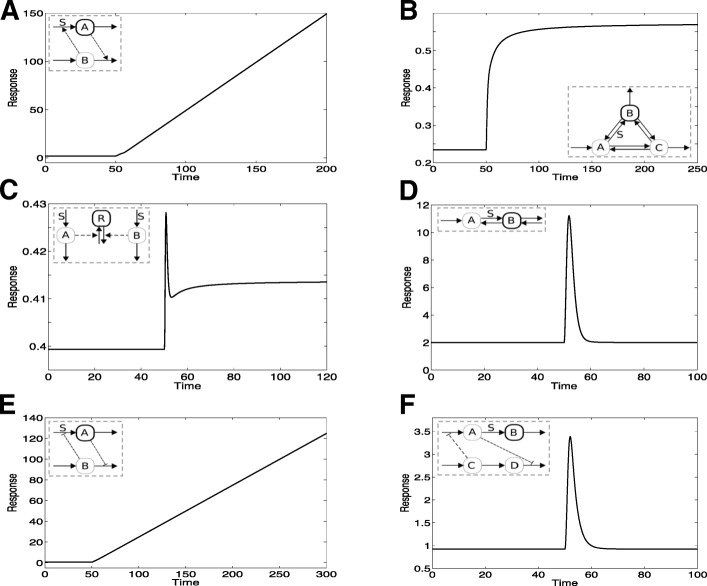
Responses of circuits to a linear ramp. A linear ramp elicits a range of qualitatively different responses in adaptive circuits such as (**a**) An unsteady state, increasing response (circuit DR12.M1: an inflow controlling open system) (**b**) A non-adaptive steady state (circuit DR08.M31) (**c**) Partial adaptation (KR11) (**d**) Exact adaptation (DR08.M1). (**e, f**) Two apparently similar looking circuits such as DR12.M4 and DR08.M1** (shown in Fig. 1) give contrasting responses. The circuits are depicted as insets in these (and subsequent) plots

**A range of contrasting responses to ramp stimuli are elicited from adaptive circuits.** We start by examining the response of these circuits to a linear ramp input. The model is subject to a low steady basal level of input, to which it adapts. Using the steady state as the initial condition, a ramp input of fixed slope is applied at a specific time *t*=50. The range of responses is seen in Fig. 2. Some circuits (such as DR12.M1) do not reach a steady state, as seen in the output of the circuit steadily increasing. Other circuits such as DR08.M31, reach a steady state but the response is non-adaptive (i.e monotonic and saturating). In fact the new steady state depends on the gradient of the ramp. In contrast other models such as KR11 exhibit partial adaptation: as seen in Fig. 2, and depending on the parameters of the model, could exhibit underadaptation or overadaptation. Finally other circuits, such as DR08.M1 exhibit perfect adaptation in a ramp, and this feature does not depend on the gradient of the ramp. This demonstrates that while all circuits exhibit perfect adaptation or partial adaptation (often close to perfect adaptation) in step stimuli, their response to temporal gradients can be strikingly different, spanning a range of outcomes.

Figure 2e,f contrasts the behaviour of apparently similar motifs *DR*08.*M*^∗∗^ and DR12.M4, both involving an open systems structure: the former adapts perfectly while the latter is very close to perfect in a step stimulus (Additional file 1: Figure S2). Their response to ramp stimuli demonstrates a clear qualitative difference: one adapts perfectly while the other does not even reach a steady state. This clearly demonstrates that ramp stimuli can elicit qualitatively different responses, and consequently be used as a basis for disciminating between adaptive circuits, even ones which appear structurally similar.

**Fig. 3:**
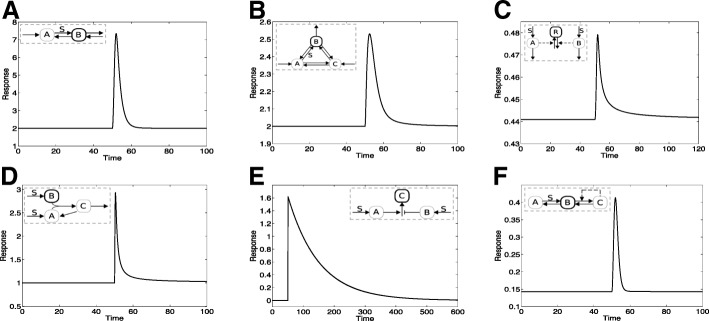
Adaptive responses of circuits. A range of circuits exhibit exact adaptation in a ramp, such as (**a**) A linear motif with inflow and outflow DR08.M1 (**b**) A cyclic motif with inflow and outflow DR08.M33 (**c**) An incoherent feedforrward motif (KR09) (**d**) An incoherent feedforward motif which can exhibit fold-change detection KI14 (**e**) An incoherent feedforward motif CO09.M2, which has (opposite) thresholds associated with each leg. (**f**) A transcritical circuit TC. The respective network motifs are depicted in the inset

**Exact adaptation to ramp stimuli.** Figure 3 shows the response of six different circuits, indicating that they all exhibit exact adaptation in a ramp stimulus. This indicates that exact adaptation can occur in response to dynamic stimuli and this is not an isolated occurrence with multiple circuits exhibiting this behaviour. Furthermore, this behaviour is independent of parameters (unless otherwise noted).

**Open Systems.** The first two circuits (Fig. 3) are those of open systems: the first having a linear topology and the second a cyclic topology. In these models the requirement that inflow matches outflow (which has to hold at steady state) results in the output adapting to a step input independent of the value of the signal. In the linear motif, when subject to a ramp, the increasing value of this signal effectively short circuits the associated step in the circuit: thus the circuit behaves as if the extra step is not present (i.e. inflow applied directly to the outflow variable) and consequently exhibits exact adaptation. Analytical results consolidate this intuitive result. A similar situation is observed in the cyclic motif. It is then worth asking, under which conditions adaptation occurs in a ramp in such inflow-outflow circuits.

**Fig. 4:**
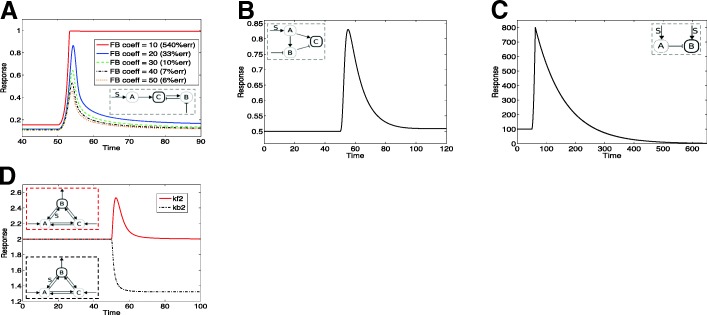
Inexact adaptation in ramp stimuli. A range of circuits may exhibit inexact adaptation in a ramp such as (**a**) A feedback motif (MA09.FB), where an increase of feedback strength brings steady state output closer to pre-stimulus value. (**b**) A feedforward motif (MA09.FB) with saturation (**c**) Another feedforward motif (CO09.M1). Here due to thresholds in one feedforward leg, the steady state reaches 0. (**d**) The location at which a signal appears in a motif can be of great importance: shown are two different locations of signal appearing in model DR08.M33, one resulting in exact adaptation and the other, a nonadaptive response. In contrast to the previous cases, the non-adaptive behaviour here is not due to saturation, and the steady state carries information about the gradient of the ramp (see text)

**Design principles associated with ramp adaptation in open systems.** Our consideration of a range of linear and cyclic motifs, with inflow and outflow reveals the following insights. (i) For linear motifs, exact adaptation in a ramp occurs, as long as the ramp signal is not associated with the conversion/degradation of the output species. If in the two species linear motif, the ramp signal was associated with the conversion of the output species, a steady state is still observed, which is not adaptive. (ii) For three species cyclic motifs, with only one outflow (the adapting variable), exact adaptation in a ramp occurs, as long as the ramp signal is not associated with the conversion of the output species to another species (also see Fig. 4, which shows how location of a signal in a network can determine the behaviour). This consolidates the insight from the previous point, incidentally. (iii) When more than one outflow variable is present, additional restrictions occur. Firstly if all species are associated with reversible reactions, adaptation does not occur in a ramp (or for that matter in a step). Adaptation is possible if some of the reactions between species are irreversible. If the additional (non-output) outflow variable is associated with irreversible reactions, then adaptation in a ramp occurs only if the signal is not associated with the interconversion from or between outflow variables. In general, greater the number of outflow variables, greater are the constraints on where the signal can act to elicit exact adaptation in a ramp. These insights emerge from analytical results discussed in the next section.

**Incoherent feedforward motifs.** Three of the motifs in Fig. 3 are incoherent feedforward motifs. The first motif is a motif used to explain adaptation in chemotaxis in Dictyostelium. Here we find that the output of the model adapts to a ramp even though some entities in the circuit do not even reach a steady state. The reason for adaptation in this circuit is the cancellation effect of two pathways, neither of which adapts, or even reaches a steady state. Since the two pathways constitute the opposing enzymes in a covalent modification cycle, the output does reach a steady state. This can be seen explicitly analytically (also see [52]) and is discussed in the next section. This feature is shared by the second feedforward motif, KI14. Another incoherent motif CO09.M2 also reveals exact adaptation in a ramp. Here the reason for adaptation is subtly different: the adaptive circuit involves (competing) pathways each associated with a threshold: their product regulates the output. Here, under basal conditions, one of the two pathways is at a zero steady state, while an increasing signal such as a ramp ends up making the other pathway fall below its threshold, again resulting in a zero steady state. This ensures that the product of the two pathways is still zero, leading to exact adaptation. This suggests how incorporating threshold effects in different “directions" in interacting/cooperating pathways will lead to adaptation in a ramp. Taken together, there are a subset of incoherent feedforward motifs which maintain a “cancellation" effect of the two pathways (realized in different ways), in increasing stimuli.

Figure 3f demonstrates that a circuit of adaptation relying on a transcritical bifurcation (TC), also results in exact adaptation. The reason why this circuit exhibits exact adaptation is different from the ones above. Here the application of a ramp results in the moving of all the species from one part of the pathway to the covalent modification cycle involving the autocatalytic feedback (which is the core of the adaptive circuit). This subsystem reaches a steady state which does not depend on the total amount of species, as is seen analytically below, explaining the adaptive behaviour (a similar result would apply to other circuits exhibiting absolute concentration robustness). Note that this depends on the location of the signal relative to core autocatalytic circuit. If the signal was appearing “downstream" of the autocatalytic species, it would not result in exact adaptation in a ramp, since this would result in the movement of species away from the autocatalytic circuit. The underlying insight can be extended to other circuits which exhibit absolute concentration robustness (discussed later): circuits whose output do not depend on the total concentration of substrate species, when “connected" to an ambient network regulated by a signal, can give rise to adaptation in a ramp though this places restrictions on the locations of the connection (and action of the signal).

Figure 4a shows the response of a feedback motif to a ramp. Here, a low feedback may result in a non-adaptive response, but a higher feedback will result in a partially adaptive response. Other examples of inexact adaptive behaviour are also shown in Fig. 4. It is worth briefly contrasting this with the behaviour of a 3 node motif DR08.M4, which is a core part of a negative feedback mechanism used to describe aspects of chemotactic adaptation in E.coli (see Additional file 1). Here the motif contains a pair of reversible reactions (with which are associated chemoattractant and chemorepellent signals). Here we find that for a ramp signal associated with one of the reactions, exact adaptation ensues, but this is not the case for the other reaction (see Additional file 1). This motif while a core aspect of a negative feedback mechanism for adaptation, actually shares many features with the inflow-outflow system models studied above (though it is a closed system), including the underpinning reason for adaptation (it could thus be included in either category). Finally looking back to Fig. 2e, we also find that nonadaptive unsteady state responses in a ramp may be seen when the signal is associated with an inflow, even with feedback: in this case, an adaptive (though not exact) response is seen in in step stimuli. Taken together, this shows how even in feedback circuits, the presence of other characteristics (zeroth order reaction, or signal associated with inflow), can significantly impact qualitative behaviour

**Fig. 5:**
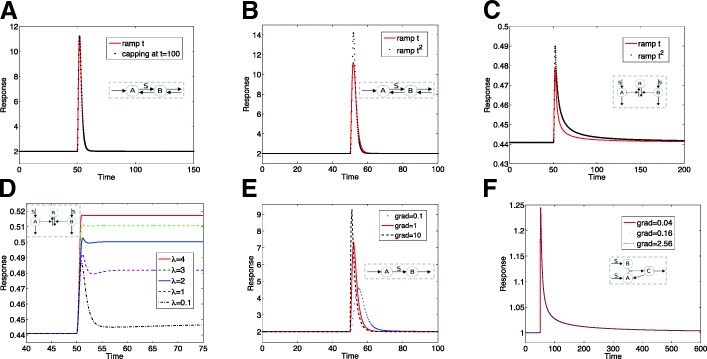
Effect of variation of the input stimulus. **a**:The effect of “capping” the ramp stimulus: DR08.M1 adapts exactly for the capped ramp signal. The transient response in this case (for this level of capping) is also practically identical to that where there was no capping of the ramp signal. **b**,**c**: A quadratic ramp:DR08.M1 and KR09 respectively adapt exactly to a quadratic ramp stimulus. In both cases the transient responses differ from that for the linear ramp signal. **d** An exponential stimulus can elicit imperfect adaptation in a circuit, even when there is exact adaptation in linear and quadratic ramps. The sensitivity of motif KR09 to the exponent is depicted. **e** The gradient of the ramp has a clear effect on the transient response, without altering the feature of exact adaptation(shown for circuit DR08.M1*). **f** While both basal level and ramp gradient can affect the response, we find that a proportional change of both keeps the response unaltered in the circuit KI14 which exhibits fold adaptation

**The effect of capping a ramp.** A ramp is an unbounded stimulus while in cellular systems there are multiple factors which result in signals being bounded. Figure 5a shows the effect of “capping" of a ramp for a circuit which exhibits exact adaptation in a ramp revealing that, the capping has no effect since the output of the circuit has already adapted. In fact, for all the circuits showing exact adaptation in a ramp (in Fig. 3), (i) exact adaptation continues to hold good (this behaviour arises from the intrinsic characteristrics of the circuit, without requiring capping) (ii) depending on the balance of level of capping and the ramp slope (high enough capping/not too steep ramp), the capping can have negligible effects on the temporal profiles as well. In other cases, capping a ramp can convert an inexactly adaptive or even a non-adaptive response to an exactly adaptive one.

**Other increasing stimuli.** We also examined other ramps (quadratic, exponential). We expect the same insights arising from the analysis above, to carry through to a quadratic ramp, or even an exponential stimulus. The one type of motif where it is not clear a priori what the response would be is the incoherent feedforward motif which relies on adaptation through cancellation of contributions of two pathways. Here, the output of such a model (KR09) adapts even to a quadratic ramp (Fig. 5). The fact that the “cancellation of pathways" works even for this stimulus can be seen analytically as discussed below. However for an exponential stimulus, we now find deviations from exact adaptation in this model (Fig. 5d) (both these features are shared by the feedforward motif KI14, see Additional file 1). Increasing the exponent of the stimulus leads to more pronounced deviations from exact adaptation, eventually resulting in non-adaptive responses. Another example of the subtle roles of the stimuli comes up in examining inflow-outflow circuits: we have already discussed how a stimulus applied to certain reactions can result in non-adaptive steady state responses. Interestingly, in such cases if the stimulus is a quadratic stimulus, this results in a zero steady state. This is discussed subsequently.

**Summary.** Our study of ramp stimuli demonstrates the range of responses which may be observed. In particular it reveals design features of circuits which enable exact adaptation in a ramp, and scenarios where non-adaptive behaviour may be observed. The implications of this, and the effect of network location and augmentation of circuits therein, are discussed in the “Discussion” section.

### Temporal periodic stimuli

We now turn to periodic stimuli. At the outset we note a basic characteristic of the response of adaptive circuits. If the period of oscillations is large (relative to the time scales of the adaptive circuit), the output remains practically unchanged: this is because this scenario corresponds to a quasistatic modulation of the input, and the output adapts to the slowly varying stimulus and is consequently practically unchanged. On the other hand if the stimuli is of high frequency, the output is again close to steady: this follows from the fact that the circuit effectively samples the average of the stimulus. We focus on scenarios which do not correspond to either extreme case. We consider a stimulus of the form *S*=*a*+*bsin*(*wt*) : a is the basal level and b is the amplitude.

**Fig. 6:**
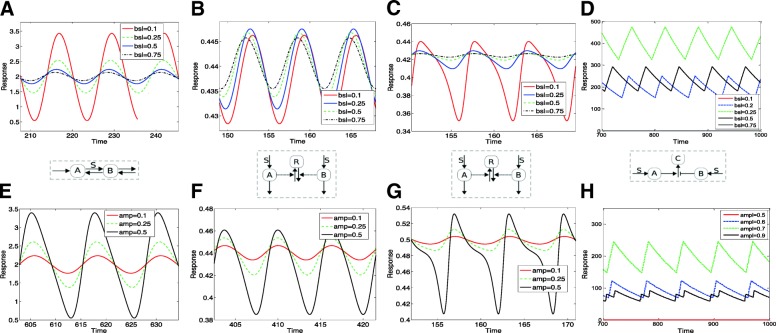
Response to periodic stimuli A range of responses to periodic stimuli are depicted. (**a**-**d**): Responses of models to a periodic signal with a fixed amplitude and varying mean value. The typical response is one where upon increasing basal(mean) value the amplitude of oscillations of the response decreases. In (a), DR08.M1 shows a maintenance of the mean of the output (explained analytically). In (**b**, **c**) KR09 and KR11 behave typically and the mean is not maintained. The effect of saturation is seen in the difference between (**b**) and (**c**). In (**d**), CO09.M2 behaves atypically, i.e. the amplitude of oscillations and their mean value reach a maximum and then decrease. (**e**-**h**): Responses of the models to a periodic signal with a fixed basal level and varying amplitude. The typical response is one where increasing the amplitude of the input increases the amplitude of the output. In (**e**-**g**) we see this typical behaviour but with maintenance of output mean value for DR08.M1 (**e**), while this is not the case for the other models (KR09, KR11). The saturation effect in (**g**) exhibits itself as a pronounced asymmetry of oscillations (relative to (**f**)). In (**h**), CO09.M2 behaves atypically: oscillations and their mean value reach a maximum and then decrease (the lowest and highest basal levels correspond to zero oscillations in (**d**) and (**h**))

**Effect of stimulus mean value.** We first consider the effect of varying the basal level for fixed amplitude (Fig. 6a-d). This reveals the following trends. For some of the circuits, especially those associated with no saturation, an increase in the basal level results in smaller amplitude oscillations, even though the average of the oscillations doesnt vary much. If we consider a model such as KR11, we can clearly see the effect of saturation: in this case increasing the stimulus mean value results in lower amplitude oscillations, but about a mean which either increases or decreases. The former behaviour is seen in circuits which exhibit underadaptation and the latter in circuits which exhibit overadaptation. A feedback motif MA09.FB shows the behaviour similar to an under-adaptive feedforward circuit. A distinct pattern in seen in the feedforward model CO09.M2. Here changing the mean value of the input stimulus causes a transition from non-periodic behaviour to periodic behaviour, whose amplitude increases, then starts to decrease, following which oscillations are lost. This illustrates how core characteristics of the circuit are brought to the fore in dynamic stimuli and result in distinct responses.

**Effect of variation of stimulus amplitude.** When the amplitude is increased, keeping input mean value fixed (a constraint on the amplitude is implicit here, since the input has to remain positive), higher amplitude oscillations are seen in multiple inflow-outflow circuits, irrespective of topology, (Fig. 6) and other circuits (Additional file 1). In some cases this can also result in a pronounced asymmetry of oscillations (even though the input is symmetric), especially when one or the other pathway saturates, as seen in the model KR11. Even in a feedforward motif without saturation, changing the amplitude also alters the mean value of the output. Finally in the case of the circuit CO09.M2, there is a transition from no oscillation to oscillations of increasing amplitude (and mean), before this decreases, again showing how intrinsic circuit features are brought to the fore.

**Variation of both mean value and amplitude.** We also studied the effect of variation of both basal level and amplitude keeping their ratio fixed (Additional file 1: Figure S7). A new notable feature is that the circuit KI14 shows no change in the response, and this factor can be traced to the fact that this circuit exhibits fold-adaptation. This can also be understood analytically.

**The variation of the output mean value.** Exact adaptation to constant stimuli means that the output steady state is independent of the stimulus level. When we consider time-varying stimuli such as periodic stimuli, we note that the the output is also a time-varying periodic stimulus. It is then worth asking, to what extent one can expect an effect like adaptation here. There are two kinds of adaptation one can think of: (i) the mean of the output is maintained irrespective of the input (mean as well as oscillation characteristics) (ii) the mean value of the input does not affect the output. In either case, we require an insulation of a mean or its effects, either from the input or the output end. If we require that the output characteristics are independent of the input mean value, we find that none of the circuits strictly meet this criterion, though some circuits exhibit a relatively modest change in output amplitude for a substantial change in input mean value.

**Design features underlying maintainence of output mean value.** We find that two classes of models show an independence of output mean value on characteristics of the input. One is the class of inflow-outflow models studied in [30]. The other circuit exhibiting this behaviour is the transcritical circuit. Robust exact adaptation in constant stimulus is associated with the presence of an integral control action. In the case of these circuits, there is the presence of an integral controller with fixed coefficients (even when the stimulus is time varying). A basic analysis reveals that in such a case the mean value of the output is maintained at a constant value independent of the input characteristics. This is discussed further in the next section. In the case of the inflow-outflow motifs, further insights can be obtained. If there is only one outflow variable, then this is seen (as long as periodic solutions are seen in the system). If there is more than one outflow variable, then there are restrictions on where the stimulus may act, for this behaviour to occur. Interestingly these restrictions only partially overlap with the restrictions on signal location for exact adaptation in a ramp.

**Summary.** Time-periodic stimuli typically elicit oscillatory responses from adaptive circuits, whose features depend on input characteristics, with the effect of adaptation reflected (though not exactly) in the mean of the output, in many cases. The exact maintenance of the mean in some circuits (with the associated design features) and the abrogation of a periodic response in specific circuits are notable points

### Spatially varying stimuli.

Thus far, we have focussed on responses to adaptive circuits to dynamic stimuli, studied in purely temporal terms. It is well known that spatial factors can have significant effects on cellular information processing. In some cases spatial aspects are of direct importance because cells have to respond to spatially graded cues (as in eukaryotic chemotaxis), while in others spatial organization of information processing can affect the temporal response, in a way which cannot be understood through purely temporal models. The effect of homeostatic mechanisms at the tissue level, in response to spatially graded signals is also relevant here. We perform an extension of some essential insights developed above to the case of spatially varying stimuli. Here, we will focus on three types of circuits above: a sample incoherent feedforward motif KR09, a three node motif with inflow and outflow DR08.M34 and the transcritical circuit. All these circuits exhibit exact adaptation in temporal ramp stimuli, and the last two circuits maintain a mean value of the output in periodic stimuli. We ask the question: what implications does this characteristic behaviour have for stimuli with spatial and temporal variation?

To consider spatial stimuli we study the spatially extended adaptive circuits in one-spatial dimension with periodic boundary conditions. This is sufficient for the insights which we draw, which are relevant in other settings (and other boundary conditions) as well. The analysis we perform is relevant both at the cellular level (adaptive response to spatially graded stimuli) and the tissue level (homeostatic mechanisms response to spatially varying stimuli, with cells stationary in the tissue).

We focus on experimentally relevant spatial analogues of the stimuli considered earlier. We examine four types of stimuli: (a) A static spatial signal. (b)A spatially homogeneous basal signal upon which is imposed a ramp stimulus whose gradient is spatially varying (c) A travelling wave (d) A standing wave. These stimuli combine dynamic characteristics of stimuli studied so far, with non-trivial spatial aspects, and can be used to probe new aspects of the adaptive/homeostatic behaviour.

**Fig. 7:**
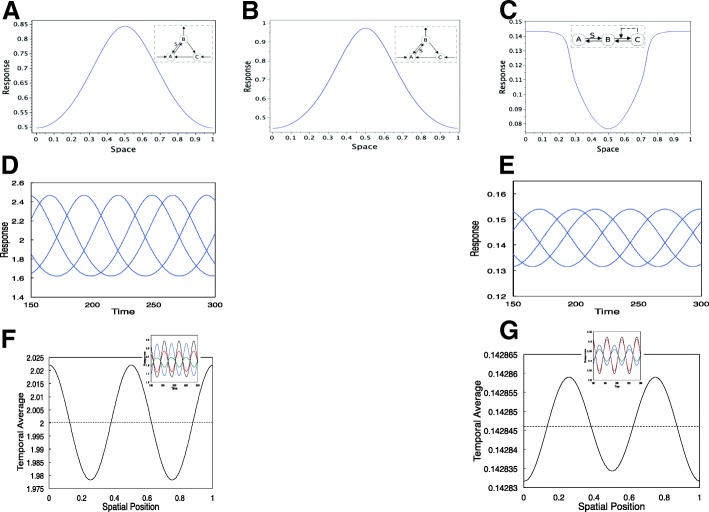
Inputs with spatial and temporal variation. (**a**) shows how an adaptive circuit DR08.M34 (where the signal converts B to A) can sense and respond to a spatial gradient which is not static in a persistent way, even without any diffusible entity. The response of two circuits: DR08.M34 and TC to ramps whose slopes vary spatially (**b**, **c**), travelling wave inputs (which elicit travelling wave responses implying that local time averaged outputs do not vary spatially)) (**d**, **e**) and standing wave inputs (**f**, **g**) are depicted. (**f**, **g**) depict the temporal average over a cycle of oscillations as a function of spatial location. In model DR08.M34 the temporal average varies with the spatial location, though the spatial average of the temporal average is fixed. TC, even with the autocatalytic species moderately diffusible, exhibits an essentially exact maintenance of temporal average at every location. The insets in (**f**, **g**) show the oscillations at different spatial locations, indicative of a standing wave.In particular (**d**, **f**) shows how a circuit can exactly maintain its local time averaged response to a travelling but not standing wave. See text for discussion

**Case 1: No species diffusible.** In this case information processing is purely local and all the behaviour studied earlier continues to hold good. We focus briefly on one case (Fig. 7a), a three node motif with inflow and outflow:DR08.M34). We found in earlier analysis that if a ramp stimulus was applied to the conversion of *B* to *A*, the system would exhibit a non-adaptive response reaching a steady state. Now if a ramp stimulus is imposed on the system with a spatially varying gradient, the adaptive circuit will give rise to a spatially graded steady state which is not adaptive (Fig. 7a). The significance of this is the following. It is usually assumed that adaptive circuits cannot be consistent with non-adaptive behaviour (gradient sensing) to spatial gradients, unless some species in the system is diffusible. We find here that a circuit can indeed adapt to a static temporal stimulus (eg a step), and be capable of preceiving spatial gradients when they are not steady, even with no diffusible species. Note that in this circuit if the signal was a quadratic ramp, this behaviour would be lost, as the steady state would be zero (discussed above).

**Case 2: Diffusible species in circuit.** In the incoherent feedforward motif KR09, it has been shown that having a diffusible species can give rise to adaptation with spatial sensing, and that differences in diffusivity can be used to achieve different combinations of temporal and spatial responses (see [8] where the model was formulated and also [34–36, 53]). In the context of the three node motif with inflow and outflow, it can easily be seen that having species *A* diffuse can give rise to non-adaptive behaviour in static spatial gradients: the essential insight being that the diffusion term contributes an extra “sink" which along with outflow has to match inflow to the system. Since the diffusion term contains spatial information (see Appendix), this means that matching inflow and outflow to the full system, will result in the adaptive variable *B* containing gradient information. In the case of the transcritical circuit, with a diffusible species *A*, the steady state of the system is one where the autocatalytic species *C*=0. This allows for a non-adaptive response of the adapting variable *B* (a non-zero steady state for the autocatalytic species is the basis for adaptation in this circuit: see analysis in Additional file 1 which shows that this is prevented in this case). Overall, having a diffusible species in the circuit can allow the species to exhibit clear gradient response (non-adaptive behaviour) in a static spatial gradient. We point out that only certain choices of diffusing variables will allow for this in general. We further note that in the case of the inflow-outflow circuits such as DR08.M34 (if *A* is diffusible), the spatial average of the output can be maintained at steady state, irrespective of the input characteristics, even while a graded response is achieved. This is true if there is only one outflow variable, and in some restricted cases when there are two outflow variables (Additional file 1). This is not the case in the other circuits.

**Temporally varying signals.** We now focus on temporally varying signals. When subject to a ramp stimulus whose gradient varies with space, all the circuits exhibit non-adaptive behaviour (Fig. 7b,c). This is not surprising noting that the same thing happens even in a steady gradient. This shows how in such cases all the circuits can give non-adaptive behaviour in such spatiotemporal ramps, even though they adapt in purely temporal ramps.

When we consider periodic stimuli, we ask if the (temporal) mean of the adapting variable is maintained, as was seen in the inflow-outflow circuits and the transcritical circuit in the purely temporal case. If no species diffuses, information processing is purely local and the temporal mean is maintained at the same value everywhere. Note that this happens even in a standing wave, where different locations are associated with different signals.

When some species are diffusible, matters are more subtle. For the transcritical circuit with species *A* diffusing (which gives rise to graded response in a static gradient), the mean of the adapting variable is still maintained both in response to standing waves and travelling waves (Fig. 7). The essential insight is that the diffusion of *A* does not affect the analysis which led to the establishment of fixed mean of the output (in the temporal case). However, if the autocatalytic variable *C* is non-diffusible, the response of the circuit (even though time-periodic at every location) cannot always be guaranteed to be qualitatively similar to the stimulus (standing wave/travelling wave). If the autocatalytic variable weakly diffuses, a close to exact maintenance of the mean value (over a temporal cycle) can be achieved (see Fig. 7 where this is practically exact even for moderate diffusion of the autocatalytic variable). Here the output of the circuit mirrors the input.

For the inflow-outflow circuit (with species A diffusing), we find, interestingly, that the output maintains its mean value (in time) in response to a travelling wave but not a standing wave.Travelling and standing wave inputs lead respectively to travelling wave and standing wave outputs. Simulations in Fig. 7 show clearly that different locations have different mean values in response to a standing wave. The fact that this motif exhibits a fixed mean in response to a travelling wave is established analytically in the next section.

**Summary.** Our consideration of spatial systems reveals how some circuits demonstrate graded responses to static spatial stimuli with exact maintenance of the (spatial) mean value, and how some circuits exhibit exact maintenance of the (local) temporal mean value in response to spatiotemporal periodic stimuli, though this can depend on both the circuit and the specific nature of the stimulus.

### Combining different types of adaptive responses

**Fig. 8:**
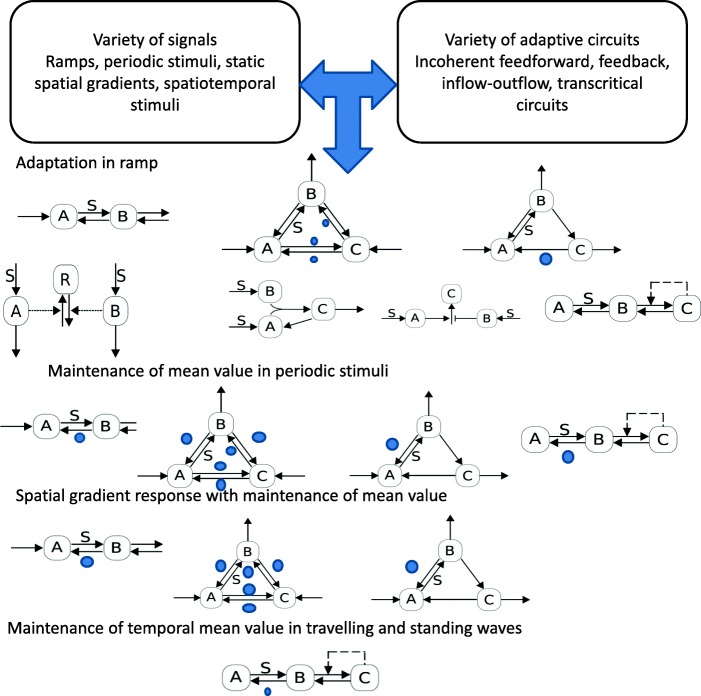
Summary of circuits exhibiting different degrees of exact adaptive responses in different stimuli. Note that the restrictions on signal locations (noted by nominal signal location and filled circles) where applicable is different for different stimuli. The spatial gradient response is realized for some species being (highly) diffusible. See text for details

**Design principles and features underpinning different kinds of adaptive responses.** Our analysis shows how different degrees of exact adaptation (parameter independent) can occur in complex stimuli: exact adaptation in a ramp, maintenance of mean value in a periodic stimulus, maintenance of mean value in a spatial gradient. We now synthesize these various results by focussing on the enabling features which make each of these behaviours possible, and how different motifs can combine one or more of these features. Adaptation in a ramp can occur in incoherent feedforward motifs, the transcritical circuit and in inflow-outflow circuits (with some restrictions). The ramp in a transcritical model acts on a step which results in the flux of species to the autocatalytic subnetwork which is responsible for this. With regard to inflow-outflow circuits we summarize the results by noting that if the adaptive variable is the only outflow variable, then a ramp will result in exact adaptation as long as it does not act on the conversion/degradation of this variable. In the 3 node network with another outflow variable more restrictions emerge. Maintenance of mean value of output in periodic stimuli, occurs in the transcritical circuit, and in inflow-outflow circuits with only one outflow. Additional outflows place restrictions on where the signal may act. The maintenance of mean value in a spatial gradient occured only in the inflow-outflow circuits, with certain species being highly diffusible. A look at all these constraints (Fig. 8) reveals, interestingly, the diverse and non-overlapping constraints placed on the circuits to achieve such special behaviour. This prompts the question as to what extent the presence or absence of these characteristic responses may be combined.

The essential results are summarized in Table 2. Here we discuss how all combinations of the presence or absence of these three classes of behaviour may be seen in different circuits as delineated in Table 2. To start with we note that inflow-outflow circuits (of linear topology for instance) where the signal does not degrade the output variable can allow for all such behaviour to be realized. The diffusion of the species associated with the first node enables the desired gradient sensing behaviour. On the other hand in multiple circuits (for example completely reversible 3 node motifs with two outflows, or even feedback models studied earlier), none of the three behaviour is observed. An inflow-outflow circuit (for eg. in a linear topology) with signal acting on the output variable would show adaptive behaviour only in periodic stimuli and in spatial gradients. We have already seen how the incoherent feedforward motifs considered result only in adaptation in a ramp, while a transcritical circuit would show adaptive behaviour in both ramps and periodic stimuli. A signal acting in the opposite reaction as depicted in a transcritical circuit would prevent adaptation in both ramps and spatial gradients. This already accounts for six of the eight possible cases, all of which directly emerge from our study of design principles. The remaining non-trivial possibilities are those which show adaptive behaviour in a spatial gradient but not in oscillations. This appears more tricky since in the models seen, adaptive behaviour in a spatial gradient occurs in a subset of models (only inflow-outflow circuits) associated with adaptive behaviour in periodic stimuli (inflow-outflow and transcritical circuits).

**Table 2:** Combinations of exact adaptive behaviour in a range of dynamic and spatial stimuli (see text for details)

Ramp	Periodic stimulus (mean adaptation)	Static spatial gradient (mean adaptation)	Circuits realizing this
Yes	Yes	Yes	Inflow outflow circuits with one outflow, restrictions on signal location (cannot act on output species)
No	No	No	Feedback circuits, Inflow-outflow circuits with multiple outflows and signal mediating reactions between outflow variables
No	Yes	Yes	Inflow Outflow circuits with one output, signal acting on output variable
Yes	No	No	Incoherent feedfoward circuits
Yes	Yes	No	Transcritical circuit (A diffusible)
No	Yes	No	Transcritical circuit (A diffusible), signal mediating conversion from B to A
Yes	No	Yes	Linear Inflow-outflow circuit with signal regulating inflow and outflow reactions, the latter through a diffusible intermediate
No	No	Yes	Same as previous case, but either with additional (nonlinear regulation of B to A reaction) or B to A conversion autocatalytic

In order to obtain the adaptive behaviour in a spatial gradient but not in a periodic stimuli, we need to consider motifs which realize certain restrictions on one behaviour but not the other. This can be done by combining characteristics of motifs. For example in a linear inflow-outflow motif, with the signal regulating both inflow and outflow (through one intermediate species, in each case, one of which is diffusible), it is possible to get adaptation in a ramp, and a spatial gradient but not in periodic stimuli (see next section). The key insight is that having a signal regulate both the inflow and outflow (one through a diffusible pathway) leads to adaptation (of mean value) in a gradient, while having a non-trivial gradient response. The adaptation of mean value in periodic stimuli does not occur, though adaptation in a ramp does occur (similar to cancellation of feedforward pathways). In order to obtain to obtain a non-adaptive behaviour in a ramp, this structure can be modified in two ways: one is to have the signal regulate the adaptive variable through conversion to the upstream species in a nonlinear way, in addition to the above regulation. Another way is to incorporate an autocatalytic effect in the conversion of the adapting species to its upstream species. In both cases, this does not affect the combination of response to spatial gradient and periodic stimulus, but results in non-adaptive response in a ramp (see next section). This accounts for the remaining two cases. This shows how our analysis of enabling design features in earlier cases can be used to construct new circuits with desired behaviour.

## Analysis of models

We now present a selection of analytical results. Our goal in this section is not to perform exhaustive analysis of all models (further studies and details are presented in the Appendix). Instead we focus on succinct analysis of a range of specific cases to clearly illuminate points made previously. This section can be skipped without any loss of continuity.

### Response to ramp stimuli

We first dissect 3 classes of basic models which exhibit adaptation in ramp stimuli: (i) Open systems with inflow and outflow (ii)The transcritical circuit and (iii) Incoherent feedforward motifs. We focus our analysis on the circuits which exhibit exact adaptation.

**Inflow-Outflow circuits:** We examined a range of inflow-outflow circuits, studied by Ruoff and co-workers. This includes a range of 2 and 3 node motifs. The analysis of response to ramps was performed in two ways: (i) studying the response (especially of two node motifs and simpler extensions to 3-node motifs) directly (ii) studying the full range of 2 and 3 node motifs using a model reduction based on a quasi-steady state analysis. Both approaches give the same results. We summarize the main insights which emerge below, with further details in the Appendix.

**Two-node motifs.** The first circuit is a two node motif with inflow and outflow. This corresponds to the model DR08.M1 (for simplicity the inflow to B is set to 0, as this does not affect any of the conclusions below). The model equations are

1 $$\begin{array}{@{}rcl@{}} dA/dt&= &k_{0} -k_{1}SA +k_{11}B  \\ dB/dt &=& k_{1}SA -k_{11}B -k_{2}B \end{array} $$

This model covers both the irreversible two node motif (*k*_11_=0) and the reversible motif.

At the outset, we note by adding the two equations that

2 $$\begin{array}{@{}rcl@{}} d(A+B)/dt &=& k_{0}-k_{2}B \end{array} $$

Thus if the system (i.e. both A and B) reach a steady state, then *B* adapts to the value *k*_0_/*k*_2_. The main insights which arise from the analysis may be summarized as follows:

Case 1 (*k*_11_=0): Here by applying a ramp stimulus *S*=*S*_0_+*S*_1_*t*, we find that *A* decays to 0 as time increases, but does so in a manner that the flux from *A* to *B* approaches a constant level *k*_0_. As far as B is concerned, the circuit behaviour essentially reduces to a one node motif with (constant) inflow and outflow, and B reaches a steady state which is of course adaptive. Further analysis is presented in the Appendix to demonstrate this.

Case 2 (*k*_11_>0): In this reversible circuit again the essential behaviour is similar to the previous case. The concentration of A approaches 0, while the net flux along the pathway from A to B approaches a constant value. Since A and B reach steady states, B of necessity will adapt to its prestimulus level. Both these examples show that the primary effect of the ramp eventually is an effective “short-circuiting" of the node A in the motif (though this insight must be carefully applied when consider reverse reactions from B to A).

Another aspect is worth mentioning when *k*_11_>0. We have associated the input stimulus as mediating the conversion from A to B. A stimulus could also have been associated with the conversion from B to A. For step stimuli applied here, the system would readily adapt based on the argument above. Interestingly however, when a ramp stimulus is applied, an important qualitative change occurs: the output reaches a steady state which is non-adaptive. The analysis of this case is presented in the Appendix. The essential insight can however be easily explained. While B reaches a nonzero steady state, the concentration of A keeps increasing. Thus *A*+*B* does not reach a steady state, and consequently *B* will not adapt exactly (if it did, it would imply that *A*+*B* reached a steady state). In fact the response is not adaptive. We make an associated point here. If the stimulus was a quadratic ramp, rather than a linear ramp: if applied in the forward direction, it would result in an adaptive response. If it was applied in the reaction converting B to A, it would result in a (non-adaptive) zero steady state response (explained in multiple ways in the Appendix).

**Three node motifs.** We now examine two three-node motifs which include the two node (reversible) motif above, and an extra node C. B is converted to C and C is converted to A. In one case there is outflow to C (DR08.M32) and in the other B is the sole outflow variable (DR08.M34). We note that when we present equations for models in the main text below, for the purposes of analysis, these include constants associated with every transition, to facillitate model analysis: when a signal is involved in a transition, it appears multiplicatively. The models presented in the Additional file 1 in some cases associate certain transitions with signals explicitly and correspond exactly to how the model is simulated. The model for the first scenario (two outflow variables) is

3 $$\begin{array}{@{}rcl@{}} dA/dt&= &k_{0} -k_{1}SA +k_{11}B +k_{31}C \\ dB/dt &=& k_{1}SA -k_{11}B -k_{2}B -k_{32}B  \\ dC/dt &=&k_{32}B -k_{33}C -k_{31}C \end{array} $$

A ramp associated with the A to B conversion leads to exact adaptation, but this is not the case when it is applied to the B to A reaction (for exactly the same reasons in the two node motif above). A ramp applied at other locations does not lead to exact adaptation of B (this is no surprise, since a step input at these locations does not lead to exact adaptation either).These results are seen through detailed analysis in the Appendix.

Now we examine a case of a 3 node motif, where B is the sole outflow variable (DR08.M34), where there is inflow to C (but no outflow: the only difference with the model above):

4 $$\begin{array}{@{}rcl@{}} dC/dt &=&k_{32}B +k_{3} -k_{31}C \end{array} $$

Here, a ramp input applied to the reactions not involving the degradation of B (i.e. A to B or C to A conversions) does lead to exact adaptation, while a ramp input applied at reactions involving degradation of B leads to a non-zero but not adaptive steady state. The reasons for this are identical to that of a 2-node motif as discussed above (see Appendix).

The Appendix presents a detailed analysis of 3 node motifs with both one and two outflow variables, and different degrees of reversibility in reactions involving the node C (note that in both the cases above C is involved only in irreversible reactions). This analysis reveals exactly what constraints emerge to satisfy exact adaptation in a ramp when reversible reactions involving C as well as multiple outflow variables are present.

**The Transcritical circuit.** The core model of the transcritical circuit (TC) involves 3 species A,B,C. The conversion from B to C is mediated by an autocatalytic feedback involving C. The model for this circuit is given by

5 $$\begin{array}{@{}rcl@{}} dA/dt&= & -k_{1}SA +k_{2}B  \\ dB/dt &=& k_{1}SA -k_{2}B -k_{3}BC +k_{4}C  \\ dC/dt &=&k_{3}BC -k_{4}C \end{array} $$

The conservation condition leads to an equation *A*+*B*+*C*=*X*_*t*_, a constant. Analysis of this network shows two distinct steady states *C*=0,*B*=(*k*_1_*SX*_*t*_)/(*k*_1_*S*+*k*_2_),*A*=(*k*_2_*X*_*t*_)/(*k*_1_*S*+*k*_2_) and *B*=*k*_4_/*k*_3_,*A*=*k*_2_*k*_4_/(*k*_3_*k*_1_*S*),*C*=*X*_*t*_−*A*−*B*. It is clear that the second steady state is physically feasible only when *X*_*t*_>(*k*_4_/*k*_3_)(1+*k*_2_/*k*_1_*S*). In this regime (which we assume, which places a lower bound on the signal) however B exhibits exact adaptation independent of S, when S is a constant. Thus exact adaptation to step increases of stimuli, naturally follows.

A ramp stimulus (mediating conversion of A to B) has the effect of converting all the A to B, so that at steady state *A*=0, whereas *B*=*k*_4_/*k*_3_ and *C*=*X*_*t*_−*k*_4_/*k*_3_. Thus, we see that B exhibits exact adaptation to a ramp. Both quadratic ramps and exponential signals also results in exact adaptation for the same reason.

**Incoherent feedforward motifs.** We turn to incoherent feedforward motifs, which, as seen earlier exhibit adaptation in a ramp stimulus. For specificity we focus on one incoherent feedforward motif (KR09) described by

6 $$\begin{array}{@{}rcl@{}} dA/dt =k_{a}S-k_{-a}A  \\ dI/dt =k_{i}S-k_{-i}I  \\ dR^{*}/dt =k_{f}A\left(R_{T}-R^{*}\right)-k_{r}IR^{*} \end{array} $$

In this model, when the signal is constant, both activator A and inhibitor I reach a steady state proportional to S. The output *R*^∗^ reaches a steady state *R*_*T*_(*A*/*I*)/(*k*_*r*_/*k*_*f*_+*A*/*I*). Since *A*/*I* is independent of S, the system adapts exactly to a step. When subject to a ramp both A and I increase without bound (if saturation is introduced as in model KR11, that will no longer be the case). Asymptotically A and I exhibit linearly increasing dependence on time with a proportionality factor which depends on the slope of the ramp: *A*∼*k*_*a*_*S*_1_*t*/*k*_−*a*_,*I*∼*k*_*i*_*S*_1_*t*/*k*_−*i*_ (*S*_1_ is the ramp slope) The output reaches a quasi-steady state, which as seen above depends on *A*/*I* and is exactly as the basal adaptive steady state. A similar adaptive behaviour is seen in a quadratic ramp for the same reason. On the other hand when the model is subject to a stimulus *exp*(*at*), then both *A* and *I* show exponential variation and *A*/*I* reaches a steady value which is not the prestimulus value. Thus the system does not exhibit perfect adaptation, and the higher the exponent is, the further the deviation from the prestimulus value. This is discussed in the Appendix. The incoherent feedforward mdoule KI14, exhibits very similar trends as this model (see Additional file 1).

### Response to periodic and spatial stimuli

While discussing the response of adaptive circuits to periodic stimuli, we highlighted two types of circuits whose response showed a mean value which was independent of the periodic stimulus. We present relevant analysis here to support those observations.

One class of circuits which demonstrate this property are inflow-outflow circuits. We first consider the two node motif with reversible interconversion studied above (this covers the case of irreversible conversion). Since *d*/*dt*(*A*+*B*)=*k*_0_−*k*_2_*B*, and the input stimulus is periodic with period T, integrating both sides over a time period

7 $$\begin{array}{@{}rcl@{}} \int_{t}^{t+T} (d/dt (A+B)) &=&k_{0}T -k_{2} \int_{t}^{t+T} Bdt \end{array} $$

Noting that the integral of the left hand side is zero, since all variables oscillate periodically,

8 $$\begin{array}{@{}rcl@{}} (1/T)\int_{t}^{t+T} Bdt &=& k_{0}/k_{2} \end{array} $$

Thus the average of B is maintained in a periodic stimulus, irrerspective of the basal value and amplitude of the input. Incidentally, even if the periodic stimulus is associated with the conversion of B to A, the same result holds good for the same reason.

Now we turn to the two 3-node motifs discussed above, which differ only in whether C is involved in outflow or inflow. If the inflow in the circuit is A and C and the outflow in B (DR08.M34), then we have

9 $$\begin{array}{@{}rcl@{}} d/dt(A+B+C)&=& k_{0}+k_{3} -k_{2}B \\ (1/T)\int_{t}^{t+T} B&=& \left(k_{0}+k_{3}\right)/k_{2} \end{array} $$

Just as before the mean value of B is maintained at the steady state adaptive level. Finally if there is outflow of C (model DR08.M32), then we have

10 $$\begin{array}{@{}rcl@{}} d/dt(A+B+C) &=&k_{0}-k_{2}B -k_{3}C  \\ (1/T) \int_{t}^{t+T} (k_{2} B+k_{3}C) &=& k_{0} \end{array} $$

Integrating the equation for C indicates that $\int _{t}^{t+T} k_{32}Bdt= \int _{t}^{t+T}\left (k_{31}+k_{3}\right)C dt$. Since the averages of B and C are proportional and noting the equation above, we find that the average of B and C are fixed independent of the stimulus. This assumes that the stimulus is associated with the conversion of A to B or the reverse conversion. This property will not in general be satisfied if the signal were associated with the conversion of B to C or C to A.

Finally, we consider the transcritical circuit above. Rewriting the equation for C (valid as long as C is non-zero) and then integrating across a period of oscillations allows us to transparently see why the mean of B is maintainated in a periodic stimulus

11 $$\begin{array}{@{}rcl@{}} d (ln C)/dt&=& k_{3}B -k_{4} \\ (1/T)\int_{t}^{t+T}Bdt &=&k_{4}/k_{3} \end{array} $$

This clearly shows why the mean of B is maintained, as long as the stimulus is not associated with the reactions involving B and C.

**Static Spatial Stimuli.** We had asserted that in a 3-node motif, with inflow at *A*, the output maintains its mean value under certain conditions. We assume that *A* is diffusible. We consider two cases, one where there is only one outflow (DR08.M34) and one where *B* and *C* have outflow (DR08.M32).

In the former case, at steady state, adding all the equations results in

$$\begin{array}{@{}rcl@{}} \frac{\partial(A+B+C)}{\partial t}&=& k_{0}+k_{3} -k_{2}B +k_{d}\frac{\partial^{2}A}{\partial \theta^{2}}  \end{array} $$

Now the LHS is zero (steady state) and integrating across the spatial domain, we find that the diffusion term integrates to zero. Thus we are left with

12 $$\begin{array}{@{}rcl@{}} (1/L)\int_{0}^{L} Bd\theta &=& (k_{0}+k_{3})/k_{2} \end{array} $$

Thus, the spatial average of the adapting variable is maintained, even though a graded response is obtained.

If we consider the case of outflow at B and C, and repeat this we find that at steady state

13 $$\begin{array}{@{}rcl@{}} k_{2}<B>+k_{3}<C>&=& k_{0} \end{array} $$

where <> denotes spatial average. If the signal is not associated with the transitions involving C, then at steady state C is proportional to B everywhere (independent of signal). In this case the spatial average of B is maintained. Otherwise in general this will not be the case. These results mirror the analysis of response of periodic stimuli in these circuits, with the only difference being the averaging is done in space rather than in time.

**Spatiotemporal stimuli.** We previously asserted that the transcritical circuit with *A* diffusing would result in a fixed mean output in spatiotemporal stimuli such as standing waves and travelling waves. This follows immediately from the analysis above

14 $$\begin{array}{@{}rcl@{}} d (ln C)/dt&=& k_{3}B -k_{4} \\ (1/T)\int_{t}^{t+T}Bdt& =&k_{4}/k_{3} \end{array} $$

which is unaffected by *A* diffusing. The conclusion is therefore the same, with the mean of *B* being maintained in both travelling wave and standing wave stimuli (also see Appendix which examines the effect of the autocatalytic species diffusing)

Now we turn to the three note motif with inflow and outflow (focussing on the variant with one outflow DR08.M34). We have

$$\begin{array}{@{}rcl@{}} \frac{\partial(A+B+C)}{\partial t}&=& k_{0}+k_{3} -k_{2}B +k_{d}\frac{\partial^{2}A}{\partial \theta^{2}}  \end{array} $$

Inetgrating across the spatial domain (and dividing by L), and integrating across a temporal period (and dividing by T) yields

15 $$\begin{array}{@{}rcl@{}} (1/T)\int_{t}^{t+T} <B>&=& \left(k_{0}+k_{3}\right)/k_{2} \end{array} $$

Here <*B*> is the spatial average of B across the domain. In the above equation, we can interchange the temporal averaging and the spatial averaging to give

16 $$\begin{array}{@{}rcl@{}} <(1/T)\int_{t}^{t+T} B>&=& \left(k_{0}+k_{3}\right)/k_{2} \end{array} $$

Now when the input is a travelling wave, the response is also a travelling wave (as seen by simulations) so that the temporal average at every location is the same as every other location. Denoting $(1/T)\int _{t}^{t+T} B$ by *B*_0_, we find that since *B*_0_ is independent of space, the preceding equation simply implies that *B*_0_=(*k*_0_+*k*_3_)/*k*_2_. Thus the temporal average of the adapting variable is maintained at the same value at every location irrespective of the characteristics of the travelling wave stimulus. This is also seen in simulations. This analysis doesnt hold good for standing waves (since *B*_0_ can vary with position) and in fact simulations clearly show that the mean is not maintained in a standing wave. We note that when the input is a travelling wave, not only is the temporal average maintained at every location, but the spatial average asymptotically approaches a constant.

### Combinations of adaptive behaviour

In the previous section we discussed circuits which could give exact adaptive behaviour in a spatial gradient but not a periodic stimulus. This can be seen, in the circuit

$$\begin{array}{@{}rcl@{}} dX/dt&=& k_{0}A -k_{2}IX  \\ dA/dt&=&k_{a}S-k_{-a}A  \\ dI/dt &=&k_{i}S-k_{-i}I +k_{d} \frac{ \partial^{2} I}{\partial \theta^{2}}  \end{array} $$

Here at steady state *X*=*k*_0_*A*/*k*_2_*I*, and furthermore *I* is spatially homogeneous. Consequently *I*=*k*_*i*_<*S*>/*k*_−*i*_. It is easy to see that <*X*>=(*k*_0_/*k*_2_)(*k*_*a*_*k*_−*i*_/*k*_−*a*_*k*_*i*_) which corresponds to maintenance of the mean value. As we have seen, in a ramp *A* and *I* asymptotically approach *k*_*a*_*S*/*k*_−*a*_ and *k*_*i*_*S*/*k*_−*i*_ and it is easy to see from a simple analysis that *X* adapts, since the dominant contribution to the long term dynamics is given by *dX*/*dt*=*k*_0_(*k*_*a*_/*k*_−*a*_)*S*−*k*_2_(*k*_*i*_/*k*_−*i*_*S*)*X* where *S*∼*αt*. Just as in the other incoherent feedforward motif, we see adaptive behaviour in a ramp. In order to get non adaptive behaviour in a ramp, this motif can be modified, to an inflow-outflow system, similar to the two node inflow-outflow system considered above:

$$\begin{array}{@{}rcl@{}} {dX}_{1}/dt&=& k_{0}A - k_{1}X_{1}+k_{11}S^{2}X_{2}  \\ {dX}_{2}/dt&=& k_{1}X_{1} -\left(k_{11}S^{2} +k_{2}\right)X_{2} \end{array} $$

The essential insight is that this regulation of the adaptive variable (but in a nonlinear way, different from the regulation of inflow and outflow and “stronger") will prevent adaptation in a ramp.This can be established in detail analytically. An alternative way is to introduce an extra autocatalytic nonlinearity by having the reaction from *X*_2_ to *X*_1_ mediated by *X*_1_. The equations are

$$\begin{array}{@{}rcl@{}} {dX}_{1}/dt&=& k_{0}A - k_{1}X_{1}+k_{11}X_{1}X_{2}  \\ {dX}_{2}/dt&=& k_{1}X_{1} -\left(k_{11}X_{1} +k_{2}\right)X_{2}  \end{array} $$

Analysis of this model also demonstrates inexact adaptation in a ramp.

## Discussion

Adaptation is a basic and widespread characteristic of information processing in cells. The primary interest in adaptation stems from the capabilities it provides a cell in its response to the environment (for eg. in chemotaxis and phototransduction), how it allows for homeostasis, and how it allows for a distinct mode of transmission of information. It is clear that dynamic stimuli/environments may be routinely encountered in cellular contexts (and may be regarded as more representative than static stimuli), and thus any in-depth understanding of the role of adaptation and homeostasis in cellular information processing has to properly account for this. Multiple models of adaptation include generic models and context-specific aspects have been proposed and studied. In trying to obtain a systematic synthesis of the response of adaptive circuits to dynamic and complex environments, it is necessary to consider different characteristics of the dynamic environment as well as different characteristics underpinning adaptation to isolate the interplay between the two. Similar broad circuit characteristics may be combined with subtle variations in model structure, which can prove important. Consequently our analysis focussed on a suite of models drawn from the literature. We summarize the essential insights which emerge and discuss its implications for natural and engineered biology.

**Responses to ramp stimuli.** We found that ramp stimuli could discriminate between even apparently similar circuits, which exhibit essentially exact adaptation to step inputs. A whole spectrum of responses from non-steady state to steady state non-adaptive, to partially adaptive to exactly adaptive responses were seen. Interestingly a range of circuits exhibited exact adaptation to linear (and even quadratic) ramp stimuli, indicative of a broader adaptive response. In cellular systems, other factors which limit the extent of stimulus, do exist. In the case of the above circuits, exact adaptation to ramps occurs purely as a consequence of the intrinsic information processing characteristics: the capping of stimulus may contribute at most a dynamic distortion of response. For other circuits which would not intrinsically adapt in a ramp, the capping of a stimulus could be a vital ingredient to convert the response to an exactly adaptive response. Our analysis delineates design features which allow for adaptation in a linear ramp: cancellation effects maintained in certain incoherent feedforward motifs,a confluenece of incoherent feedforward and threshold effects in others, short-circuiting of steps in inflow-outflow circuits. Another distinct design feature involves the ramp transferring species in closed circuits to the core adaptive subcircuits (eg transcritical circuits), which are reminiscent of circuits exhibiting absolute concentration robustness [54] to the total amount of species in the circuit. In inflow-outflow circuits, we found that the location of a stimulus in the circuit/motif could be critically important, with some locations associated with adaptive behaviour and other not, even though all these locations were associated with exact adaptation in step stimuli.

**Responses to time-periodic and spatial stimuli.** Basic as well as subtle aspects of the underlying circuit are reflected in the dynamic response to periodic stimuli. A small selection of circuits maintain the mean value of output irrespective of change in mean value or amplitude of the input. Analysis in these cases reveals the prescence of an integral controller (integrator) with constant (time invariant) coefficients, which is responsible for this. These circuits include inflow-outflow circuits and transcritical circuits. In the former, increasing the number of outflow variables (from one), imposes increasing restrictions on the degree of reversibility in the network, and the locations where the signals act to elicit such behaviour. Finally we performed some focussed analysis to extend these insights to the spatially distributed case (both input and circuit being spatially distributed: representative of either single cell or tissue levels). Interestingly we find that some circuits are capable of detecting spatial gradients in a persistent manner, when there is a temporal gradient as well, even in the absence of any diffusible inhibitor, primarily because the response in a temporal ramp is non-adaptive. Thus the requirement of having a diffusible species is not needed for dynamic spatial gradients. Having diffusible species can allow for a circuit to give a gradient (non-adaptive) response to static gradients, though this depends on which species is diffusible. Some circuits, notably inflow-outflow circuits, exhibit this non-adaptive gradient behaviour, while maintaining their spatial mean, indicating again an adaptation “in the mean". For spatiotemporal stimuli such as travelling waves and standing waves, some circuits essentially maintain the (temporal) mean-value of the response at every location to both stimuli, while others do so for only travelling waves. This clearly shows how echoes of the precise temporal structure of adaptation are seen even in spatially extended systems, but with the nature of the spatial signal and the nature of the circuit and their interplay playing important roles.

**Exact adaptation in combinations of complex stimuli.** Our simultaneous consideration of ramps, periodic stimuli and static spatial gradients, brings to the fore the different constraints and requirements for exact adaptation in ramps, adaptation of the mean in periodic stimuli and in static gradients (Table 3). In all cases considered, the factors which give rise to exact adaptation are structural and parameter independent. We demonstrated that it is possible to construct circuits which can exhibit any combination of the prescence or absence of exact adaptive behaviour to each of these stimuli. In particular, certain inflow-outflow circuits are capable of exhibiting exact adaptive behaviour to all three stimuli, demonstrating a broad and versatile adaptive behaviour. Also worth contrasting is adaptation of the mean value in periodic and static spatial stimuli, which reveals an important but subtle difference between time and space. The circuits which exhibit adaptation of mean in both periodic and static spatial stimuli (while exhibiting non-constant behaviour) show the presence of a constant coefficient integral controller. The transcritical circuit allows for mean adaptation in periodic stimuli, but not spatial stimuli: this distinction can ultimately be traced to the fact that time appears as a first derivative while space as a second derivative in the model. On the other hand, the combination of feedforward structures, with an inflow-outflow circuit can give rise to adaptation in the mean in static spatial gradients, but not periodic stimuli.

**Table 3:** Exact adaptive behaviour of classes of circuits considered in the text to different stimuli

	Feedback	Incoherent Feedforward	Inflow-outflow	Other
Ramp	No (unless zeroth order reaction present*)	Yes (no saturation of feedforward legs), also threshold/saturation model	Yes, provided signal doesnt act on output. Further restrictions if there are multiple outflow variables	Yes
Periodic stimulus (mean value)	No (unless zeroth order reaction present*)	No	Yes if only one outflow. Restrictions on signal location for multiple outflow variables	Yes
Static gradient (mean value)	No (unless zeroth order reaction is present, not studied)	No (but see hybrid case in Table 2)	Yes for one outflow variable, for some species diffusible; further restrictions on signal location for two outflow variables	No
Spatio temporal periodic stimuli	No (not discused)	No	Travelling wave but not standing wave	Both Travelling and standing wave (almost exact: see text for details)

Note that for the purposes of this table, circuits with zeroth order reaction (apart from incoherent feedforward circuits whose legs may be regulated by zeroth order reaction), including closed systems (eg. DR08.M4), are classified with inflow-outflow circuits

It is clear that there are many variations of each class of circuit (and even an individual circuit) we have studied, along with augmentations. Our isolation of the underlying design features allows us to evaluate other such circuits (including new ones which have not yet been constructed/studied) and the consequences of augmentations/variations, though this will have to be done on a case-by-case basis.

Our analysis has been based on models of adaptation which are primarily ODE based with a focus on exact adaptation which can be understood in structural terms, independent of model parameters and their tuning (in the transcritical model alone, we have noted a broad parameter range for exact adaptation). This is valid as long as the original model description is valid, which we assume. When a ramp signal is associated with an inflow reaction, we assume this description remains valid. We recognize that inexact (but close to exact) adaptation could be just as relevant, and that biology may employ additional layers (eg thresholds) to transform inexact adaptation into exact adaptation. In such cases, the nature of the adaptive response to complex stimuli needs to be studied on a case-by-case basis, and can build on the foundation here, with additional parametric analysis.

We now discuss the relevance of our results to systems biology. Dynamic stimuli such as ramps have been used experimentally in specific contexts, such as osmoregulation, and chemotaxis:the response of E. coli to exponential ramps has been studied experimentally, as has the response of the gradient sensing network in Dictyostelium, where adaptation to linear ramps has been demonstrated [14, 46, 47]. On the other hand there are many other contexts, notably in homeostasis, where the response to dynamic stimuli has not been examined in detail. This is especially true in the case of "complex" homeostatic mechanisms involving multiple layers of homeostasis, for eg see [55]. Our study provides a platform for probing such systems, by examining the response of a variety of circuits to different classes of stimuli, and also by isolating key structural characteristics for different kinds of behaviour. Our study of ramps and periodic stimuli together, presents interesting parallels and contrasts. The response to ramps spans a broad range of behaviour ranging from exact adaptation to non-adaptive behaviour. Viewed from the perspective of homeostasis, exact adaptation and non-adaptive behaviour represent opposite ends of the spectrum, and our analysis allows us to transparently isolate the reasons for both these behaviours. Non-adaptive behaviour in a ramp, especially when exact adaptation is observed in a step input, represents a breakdown in homeostasis induced by the temporal nature of the input stimulus. In response to periodic stimulus, while exact adaptation in the mean was observed in some cases, a complete failure in maintaining the mean of the output, for instance, was rarely observed.

Our study also revealed that the location of the network where the input acted could be critical in determining whether exact adaptation was observed or not: notably in multiple inflow-outflow circuits, a change of location could completely alter the response and even make the circuit non-adaptive. The fact that this is seen even in basic 2-node inflow-outflow circuits indicates that there is a fundamental constraint of such circuits to exhibit exact adaptation to a ramp, when the signal acts at multiple network locations. This has implications for biological signalling where multiple inputs may act at different points in a network, and this suggests that there are preferred locations for a signal to act to enable homeostatic responses to such dynamic stimuli. It is also suggestive of the fact that there are nodes, which exhibit homeostatic behaviour in simple stimuli, exhibit potential "fragility" (i.e. marked departure from homeostasis) in certain classes of dynamic stimuli. This could have significant consequences for when a cell may not be able to withstand certain stressful signals (depending on the nature of the stimulus and location). It remains to be seen if such locations have been avoided in evolution, or if other factors (which have the effect of capping such stimuli) have been incorporated to limit this effect. Furthermore there are biological processes where opposite steps of the same network may be targeted to achieve opposite responses—for eg in chemoattraction and chemorepulsion. Our study suggests that there are fundamental basic constraints which create clear contrasts in the nature of adaptive response to ramp-like stimuli, showing how adaptive behaviour in ramps of both types of stimuli (attractant and repellent) may not be accommodated in such cases. Our study of multiple inflow-outflow circuits also reveals the potential consequences of augmenting a circuit with other steps: this can cause a complete alteration in the nature of the response. Since evolution in biology is believed to act by " tinkering" from existing circuits, this may create important new constraints for circuits thus constructed. On the other hand by creating an augmentation, in some cases it is possible for a different signal to act at other locations to also enable exact adaptation in a ramp: as an example, an augmentation of the two-node inflow-outflow circuits with a third node with irreversible steps (Fig. 8), allows for an input (for instance with an opposite effect, such as chemorepulsion) to act on a new node which removed the constraints of the two node circuit and enable exact adaptation in a ramp.

Spatially varying signals present a distinct aspect. Here in general adaptation/homeostatic behaviour is inherited from the network. There are many ways in which adaptation could act: (i) act at every location, where the input is present (ii) A spatial averaging is performed and the adaptation occurs downstream (iii) the adaptation and averaging operations are integrated, allowing for a non-adaptive response in spatially graded stimuli. In the last case we show how it is possible to exactly maintain the spatially averaged mean value of the response. This could be of relevance at the cellular level (spatial averaging of the output from the membrane being a trigger of downstream activity) or even at the tissue level (the spatial average of outputs from an array of cells being an input to some downstream communication, or involved in some additional developmental step). It is interesting to note that the gradient sensing network in Dictyostelium which occurs via a Local Excitation Global Inhibition module, does not in general allow for the exact maintenance of the mean value of the response (the lipid *PI*(3,4,5)*P*_3_), while a network mimicking a basic E.coli adaptive circuit, but with a diffusible entity, can do so, provided certain specific enzymatic reactions act in the unsaturated limit (this follows by analyzing such circuits which are similar to the inflow-outflow circuit we have analyzed). The new considerations which emerge when considering the interweaving of temporal and spatial stimuli is reinforced by our observation that circuits which exhibit both these features, do not maintain the mean value (in time) in spatio-temporal periodic stimuli.

Adaptation and homeostasis is an important ingredient for synthetic biology as well [56, 57]. Our insights into design features for adaptation in dynamic and spatial stimuli, lays bare key ingredients for engineering sophisticated biosensor circuits with a variety of adaptive responses in dynamic environments Given the importance of biosensors in synthetic biology, biomedicine and biotechnology, and the experimental work in this direction, adaptive and homeostatic regulators (especially in complex and dynamic environments) offers a vital capability to combine with bio-sensing [58]. Going even beyond the biological area, the advent of “soft robots" [59] which could conceivably be endowed with chemical sensing capabilities, and incorporating adaptation at the sensory level could be relevant here as well. Our insights are also relevant to the engineering of information processing and homeostatic controllers through non-enzymatic mechanisms such as DNA strand displacement reactions [60–64]. We have shown how it is possible to construct compact adaptive circuits which combine features of exact adaptation in static stimuli, ramps, temporal periodic stimuli and static spatial stimuli (or any subset of these capabilities). Our consideration of structural features which robustly enable this, as well as the effect of modular augmentation of such circuits, as well as choice of nodes at which stimuli act is relevant to both bottom-up construction as well as re-wiring of existing circuits. Finally, while design in synthetic biology focusses typically or a circuit, an alternative approach is to focus on the design of a stimulus or environment to elicit certain outcomes. Our analysis here provides insights into when this may be possible (when adaptation is present), for instance by eliciting non-homeostatic outcomes through the application of temporal and spatial stimuli. Whether engineering through synthetic biology (either conventionally or through strand displacement reactions) or even other chemical means [65], adaptation and homeostasis serves as a crucial focal point, and it can be expected that many interesting applications arising from this can be realized in the fairly near future.

## Conclusions

Our simultaneous consideration of a variety of dynamic and spatial stimuli, on one hand and a variety of adaptive circuits on the other, provides insights into the types of adaptive responses which are possible in such stimuli, what features of circuits robustly enable such responses, and when adaptation/homeostasis may be compromised. This provides a platform for understanding adaptation/homeostasis in multiple cellular contexts, which may employ such circuits, or variations or combinations thereof. It allows for the evaluation of the adaptive responses of concrete cellular systems, including the robustness of the adaptive/homeostatic mechanisms employed. Ultimately it can also provide insights into whether the nature of the dynamic environment may have impacted the adaptive circuits which have emerged in evolution. On the other hand it provides a basis for engineering adaptive/homeostatic circuits for use in complex environments either by rewiring existing circuits or building circuits ab initio.

## Appendix

### Analytical results on open systems.

In this section, we will analyze in a little more detail, 2 node and 3 node motifs with inflow and outflow, to ramp inputs, to further consolidate the points in the text. This is done in two different ways: firstly we analyze a selection of the models directly to illustrate the main insights. We then analyze this using quasi-steady state model reduction: this is done on a broader range of inflow-outflow motifs

To start with we consider the two node motif (neglecting any inflow to B):

17 $$\begin{array}{@{}rcl@{}} dA/dt&= &k_{0} -k_{1}SA +k_{11}B  \\ dB/dt &=& k_{1}SA -k_{11}B -k_{2}B \end{array} $$

We first start with the purely irreversible two node motif, i.e *k*_11_=0, for the model DR08.M1 (again inflow to B is neglected). We subject this model to an increasing signal *S*(*t*) such as a ramp. To study the eventual behaviour of this system, it is useful to change variables to *w*=*SA*−*k*_0_/*k*_1_,*B*_0_=*B*−*k*_0_/*k*_2_

Then we have

18 $$\begin{array}{@{}rcl@{}} dw/dt &=&(w/S)dS/dt- k_{1}Sw +(k_{0}/k_{1}S)dS/dt  \\ {dB}_{0}/dt &=&k_{1}w-k_{2}B_{0} \end{array} $$

Looking at the terms on the RHS of the first equation, for linear and quadratic ramps the first involving (1/*S*)*dS*/*dt* becomes small as time increases relative to the second, and the last term, which is independent of w also approaches zero. Therefore as time progresses, the system evolves to *w*=0, and it is dominated by the second term. This can demonstrated formally. Even when the signal is an exponential, while the last term approaches a constant, the second term dominates (both this and the first term) and so w approaches zero. In other words *SA* approaches *k*_0_/*k*_1_. This shows that overall A decreases in concentration so that the flux out of A (and into *B*) approaches *k*_0_. From this it easily follows that *B*_0_ approaches a steady state of zero implying that *B* approaches a steady state of *k*_0_/*k*_2_. This is consistent with the intuition that the intermediate step is effectively short circuited by the increasing signal, and explains why B adapts.

We now consider the reversible case, where *k*_11_ is nozero. The approach is similar. We employ a change of variables to *w*=*SA*−*k*_0_/*k*_1_−*k*_11_*k*_0_/(*k*_2_*k*_1_),*B*_0_=*B*−*k*_0_/*k*_2_. Here the equations in the new variables read

19 $$\begin{array}{@{}rcl@{}} dw/dt&=&- k_{1}Sw +k_{11}{SB}_{0}+[(w+k_{0}/k_{1}(1+k_{11}/k_{2}))]/S dS/dt  \\ {dB}_{0}/dt&=&k_{1}w-k_{2}B_{0} -k_{11}B_{0} \end{array} $$

As before, the asymptotic behaviour is governed by the terms linear in *w*,*B*_0_ (the first two dominate the last term in the first equation). An inspection of this matrix readily reveals that for any fixed *S* it has eigenvalues which are negative or with negative real part, and with increasing S, *w* and *B*_0_ approach zero, just as before. This demonstrates the assertion that *B* adapts in this situation as well.

Now we briefly analyze the situation where the signal regulates the conversion of B to A. This is described by modifying the above model to reflect this

20 $$\begin{array}{@{}rcl@{}} dA/dt&= &k_{0} -k_{1}A +k_{11}SB  \\ dB/dt &=& k_{1}SA -k_{11}SB -k_{2}B \end{array} $$

We now demonstrate two points (i) In a linear ramp, B will not exhibit exact adaptation (ii) In a quadratic ramp, B will reach a zero steady state. Both these observations have been seen in simulations.

To demonstrate the first point: suppose B reaches a steady state (a necessary pre-requisite for adaptation). Let us call this steady state *B*_0_. Examining the asymptotic evolution of A, we see that it is asymptotically governed by the equation:

21 $$\begin{array}{@{}rcl@{}} dA/dt&=&k_{0}+k_{11}(a+bt)B_{0} -k_{1}A \end{array} $$

where the ramp *S*=*a*+*bt* is incorporated. As a consequence of this increasing production term, A also evolves to a behaviour, which is dominated by this term. This can be seen by ignoring the *k*_0_ and *a* terms above, and seeing the evolution of A. This results in a dominant behaviour of A to be *A*∼*bB*_0_*t*/*k*_1_ (the full solution can be derived, and it is clear from that this is the dominant behaviour). Now when we consider the fact that *d*/*dt*(*A*+*B*)=*k*_0_−*k*_2_*B*, we see that with a linear increase of A in time, the left hand side contributes a constant, and hence B never reaches its prestimulus behaviour. Thus exact adaptation is not observed.

Finally we also demonstrate that in a quadratic ramp, a non-zero steady state cannot be obtained. We follow the same line of reasoning. Suppose B reaches a steady state *B*_0_. For a quadratic ramp, the asymptotic dynamics of A are described by

22 $$\begin{array}{@{}rcl@{}} dA/dt&=&k_{0}+k_{11}\left(a+bt^{2}\right)B_{0} -k_{1}A \end{array} $$

Now the domainant behaviour of the A dynamics arises from the quadratic term: in fact *A*∼*bB*_0_*t*^2^/*k*_1_ as long as *B*_0_>0. Now if we examine the overall equation *d*/*dt*(*A*+*B*)=*k*_0_−*k*_2_*B* we see that such a behaviour of A is inconsistent with a non-zero steady state for B: the left hand side has a dominant contribution which arises from a quadratically increasing function of time encoded in A. Therefore, to leading order the left hand side is a linearly increasing function of time, which is inconsistent with the right hand side which approaches a constant. The assumption that *B* reaches a non-zero steady state leads to a direct contradiction and the role of the quadratic ramp is transparently seen here.

**Three node motifs.** We concisely discuss the behaviour of two 3 node motifs presented in the text: DR08.M32 (node C associated with outflow) and DR08.M34 (node C associated with inflow). These are also presented with alternative analysis in the section to follow. In the two outflow case, the only location associated with exact adaptation in a ramp is the A to B reaction. That exact adaptation occurs in this case, follows from a simple extension of the 2-node motif case, for the same reason. Exact adaptation does not occur for the other three locations: for a signal applied to the B to A reaction, exact adaptation of B, implies a steady state and exact adaptation of *C*=*k*_32_*B*/(*k*_31_+*k*_33_), but linearly increasing A. This contradicts the mass balance, *d*/*dt*(*A*+*B*+*C*)=*k*_0_−*k*_2_*B*−*k*_33_*C*, since the RHS =0 for exact adaptation, while the LHS is non-zero. In this motif exact adaptation does not occur in a step in the B to C and C to A reactions, and this is also the case in a ramp.

In the one outflow motif (DR08.M34), a ramp results in exact adaptation, as long as it is not applied to the B to A and B to C reactions. We focus on the B to C and C to A reactions (reactions introduced by the third node). In the former case, exact adaptation of B implies linearly increasing C and A with time, asymptotically, which contradicts *d*/*dt*(*A*+*B*+*C*)=*k*_0_−*k*_2_*B*: the LHS >0 while the RHS =0. For a ramp applied to the C to A reaction C approaches a zero steady state, while the influx to C (from both B and the inflow) matches the flux from C to A. This scenario is analogous to having an additional source of inflow to A and an additional (constant rate) reaction from B to A (in a 2-node motif)

**Alternative analysis of inflow-outflow circuits.** We now present alternative analysis of inflow-outflow circuits for ramp inputs. This is based on quasi-steady state approximations for some species. In particular we demonstrate through this approach that (i) For a two-node reversible motif, exact adaptation ensues if the ramp is applied to the conversion of A (the inflow variable) to B (the outflow variable, adapting). (ii) Exact adaptation does not occur if the ramp is applied to the interconversion of B to A (iii) For a three node motif (involving an extra node C) where C is produced by B and converts to A, we consider two cases: outflow only through B, and outflow through both B and C. In the former case, adaptation occurs as long as the ramp is not applied to interconversion reactions arising from the degradation/conversion form B. (iv) If there are two outflow variables, we primarily focus on the case of the B to C and C to A reactions being irreversible. In such a scenario, we show that only a ramp applied to the A to B reaction, leads to exact adaption. We also briefly consider reversible 3 node motifs.

**Two node motif.** Consider the two node motif, which is assumed to be reversible, without loss of generality. Suppose the ramp is applied from A to B. A ramp is associated with an increasing, large signal. This allows us to make a quasi-steady state approximation for *A* as *A*=(*k*_0_+*k*_11_*B*)/*k*_1_*S*. Using this in the evolution equation for B yields

$$\begin{array}{@{}rcl@{}} dB/dt&=& k_{1}S(k_{0}+k_{11}B)/k_{1}S -k_{2}B -k_{11}B  \end{array} $$

Thus as time becomes large, the dynamics of B approaches an evolution equation of the form *dB*/*dt*∼*k*_0_−*k*_*s*_*B* which indicates that B approaches a steady state which corresponds to exact adaptation. This is consistent with what was derived earlier indicating that *A* approaches 0 as 1/*S*.

Now we consider the signal associated with the B to A conversion. Here the quasi-steady approximation for B yields *B*=*k*_1_*A*/(*k*_11_*S*+*k*_2_). This now results in an asymptotic evolution equation for A of the form

$$\begin{array}{@{}rcl@{}} dA/dt&=&k_{0}+k_{11}S[k_{1}A/(k_{2}+k_{11}S)] -k_{1}A  \\ dA/dt& =& k_{0} -k_{1}{Ak}_{2}/(k_{2}+k_{11}S)  \\ dA/dt & \sim& k_{0} -(k_{1}k_{2}/k_{11})(A/S) \end{array} $$

In making the transition between step 1 and step two, we note that the difference between the the last two terms is calculated without making any assumption in the denominator of the second term. Note that these two terms are of comparable order, and neglecting the *k*_2_ in the denominator of the second term, will lead to an incorrect deviation. In fact once the second and third terms are combined, the assumption of large *S* is made. After substituting for a linear ramp stimulus, the last equation is the form *dA*/*dt*=*a*−*bA*/*t*, where *a* and *b* are constants. This can be solved by a change of variables *A*=*ut*. In terms of *u* the equation is *tdu*/*dt*=*a*−(*b*+1)*u*, which can be solved by separation of variables. This results in the solution [*a*−(*b*+1)*u*(0)]/[(*a*−(*b*+1)*u*(*t*)=*t*^*b*+1^]. From this, it follows that u approaches a steady state *a*/(*b*+1) (which contains information about the ramp slope) and consequently *A* linearly increases with time. This linear rate of increase with time numerically matches well results seen through computer simulations. From the expression for *B* we see that in a linear ramp, *B* asymptotes to a steady state not corresponding to exact adaptation, as seen earlier. Now suppose a quadratic ramp is applied: from the asymptotic evolution equation for A, we see that *A* will still approach a linear profile. This is because the variable *u*=*A*/*t* satisfies an equation of the form *tdu*/*dt*=*a*−*u*−*bu*/*t*, the last term can be neglected relative to the penultimate one as time becomes large. Thus u approaches a constant value and consequently *A* is linear. From this, looking at the quasi-steady approximation for B, we find that B approaches zero, exactly as seen in simulations.

**3-node motifs with single outflow** We focus on the model where the reactions involving the third node C are irreversible (for simplicity here and below we ignore inflow to C as that does not affect the qualitative conclusions):

23 $$\begin{array}{@{}rcl@{}} dA/dt&= &k_{0} -k_{1}SA +k_{11}B +k_{31}C \\ dB/dt &=& k_{1}SA -k_{11}B -k_{2}B -k_{32}B  \\ dC/dt &=&k_{32}B -k_{33}C -k_{31}C \end{array} $$

If *k*_33_=0 there is no outflow through C. This is the case we consider here. Now if we look at ramp stimuli applied to the conversion from A to B and that from B to A, we find that the former one adapts, but the latter one does not. This emerges from an identical approach to quasi-steady state approximation to the one considered about, just including the augmentation of the C variable. The C “leg" of the pathway is just an additional pathway from B to A. In fact the insights here follows essentially from the consideration of the interaction of nodes A and B. For instance, if the signal was associated with the transition from B to A, we find, by an identical quasi-steady state approximation, a steady state for B and a linearly increasing profile for A, whose slope does not depend on reactions involving C. The slope obtained from the quasi-steady state approximation matched with computational simulations.

We now consider the ramp applied to the other two transitions. First consider the ramp applied from C to A. A quasi-steady state assumption for *C* results in *C*=*k*_32_*B*/*k*_31_*S*. This reduces the model to

$$\begin{array}{@{}rcl@{}} dA/dt&=&k_{0} +[k_{32}B/(k_{31}S)]k_{31}S -k_{1}A +k_{11}B \\ dA/dt&=& k_{0} +[k_{32}B] -k_{1}A +k_{11}B \\ dB/dt&=& k_{1}A -(k_{11}+k_{2}+k_{32})B  \end{array} $$

This is just like a two node motif with an extra pathway from B to A. These equations when analyzed are simply indicative of a steady state for A and B with B adapting exactly.

The remaining case is when the signal acts from the B to C conversion. Here by applying the quasi-steady state approximation for B, we have *B*=*k*_1_*A*/(*k*_11_+*k*_2_+*k*_32_*S*). Implementing this reduction into the equations for A and C result in equations of the form

$$\begin{array}{@{}rcl@{}} d(A+C)/dt&=& k_{0} -k_{1}{Ak}_{2}/(k_{32}S)  \\ dC/dt &=& k_{1}A-k_{31}C  \end{array} $$

Analysis of this equation shows that C asymptotically is proportional to A with A approaching a linearly increasing function of time. Just as in the two node motif, B approaches a non-adaptive steady state. This justifies the statements made earlier.

We now turn to the case of a fully reversible 3 node system (depicted below for signal mediating conversion of A to B) which is described by the following equations:

24 $$\begin{array}{@{}rcl@{}} dA/dt&= &k_{0} -k_{1}SA +k_{11}B +k_{31}C -k_{13}A \\ dB/dt &=& k_{1}SA -k_{11}B -k_{2}B -k_{32}B +k_{23}C  \\ dC/dt &=&k_{32}B -k_{33}C -k_{31}C +k_{13}A -k_{23}C \end{array} $$

Again we focus on the case of no outflow of C, i.e. *k*_33_=0. From simulations we find that when a ramp is applied to any of the transitions, excluding those involving degradation/conversion of B, exact adaptation ensues. While we will not repeat all the calculations in these cases, we focus on signal acting at two transitions (i) A to C and (ii) C to B, neither of which was present in the previous case. We examine the first case. Here, from a quasi-steady state for A we have *A*=(*k*_0_+*k*_11_*B*+*k*_31_*C*)/(*k*_1_+*k*_13_*S*). Substituting, we have

$$\begin{array}{@{}rcl@{}} dB/dt &=& k_{1} (k_{0}+k_{11}B +k_{31}C)/(k_{1}+k_{13}S) -(k_{11}+k_{2}+k_{23})B \,+\, k_{32}C  \\ dC/dt &=& k_{23}B -k_{32}C +k_{3}S(k_{0}+k_{11}B +k_{31}C)/(k_{1}+k_{13}S) -k_{31}C  \end{array} $$

This simplifies to

$$\begin{array}{@{}rcl@{}} dB/dt&=&k_{32}C- (k_{11}+k_{2}+k_{23})B  \\ dC/dt&=& k_{0}+k_{11}B +k_{23}B -k_{32}C  \end{array} $$

It is easy to see (for instance by adding the two equations) that this system reaches a steady state, which corresponds to exact adaptation of B. In fact this system has the structure of a 2-node motif, with inflow through C and outflow through B, with an extraneous pathway converting B to C. Note incidentally that if there was an outflow through C (*k*_33_≠0), then exact adaptation would not ensue.

Now we examine the case where the C to B transition is mediated by the signal. Here from a quasi-steady state for C, we have *C*=(*k*_13_*A*+*k*_23_*B*)/(*k*_31_+*k*_32_*S*).Substituting, we have a reduced model

$$\begin{array}{@{}rcl@{}} dA/dt &=&k_{0}-k_{1}A +k_{11}B +k_{31}(k_{13}A +k_{23}B)/(k_{31}+k_{32}S) -k_{13}A  \\ dB/dt &=& k_{1}A \!- k_{11}B -k_{2}B -k_{23}B +k_{32}S(k_{13}A +k_{23}B)/(k_{31}+k_{32}S)  \end{array} $$

which simplifies to

$$\begin{array}{@{}rcl@{}} dA/dt&=&k_{0}-k_{1}A +k_{11}B -k_{13}A  \\ dB/dt &=&k_{1}A-k_{11}B -k_{2}B +k_{13}A  \end{array} $$

From this we see easily that an adaptive steady state is attained. In fact, this is similar in structure to a basic two node motif, but with an extra pathway between A and B. The steady state of B is the adaptive steady state balancing inflow and outflow.

Taken together, we find that when we have only one outflow, for transitions independent of the degradation/conversion from B, exact adaptation ensues.

**Two outflows.** We briefly examine the case of two outflows from this perspective. If the system is fully reversible, then exact adaptation does not ensue in a step, at any location. Thus, there is no reason to expect it to act in a ramp, and indeed this is not observed. Now if we consider the case where the reactions involving C are irreversible, we find that exact adaptation occurs only when the ramp is applied in the A to B conversion. The fact that the other locations do not result in exact adaptation can be seen as an extension of the above analysis. We also note that a step signal acting at these locations does not lead to exact adaptation either. In essence, the steady state of the full system implies that a linear combination of B and C is constant. Exact adaptation for B ensues only when the B to C ratio is fixed at steady state independent of the signal. This can happen only when the signal does not involve any reactions involving C. Combining this with the earlier analysis that the signal cannot be associated with conversion of B, we find that there is only one transition which is associated with exact adaptation in a ramp.

**Incoherent feedforward motifs.** We study the behaviour of an incoherent feedforward motif KR09 whose equations are presented earlier (similar insights follow for model KI14): We consider its behaviour in response to linear ramps, quadratic ramps and exponentials. Now suppose *S*=*a*+*bt*. The asymptotic leading order behaviour for both A and I are determined by the increasing term *bt*.A full solution is easily obtained,

$$\begin{array}{@{}rcl@{}} A(t) &=& A(0) exp (-k_{-a}t) +k_{a}bt/k_{-a} -\left[k_{a}b/(k_{-a})^{2}\right] (1-exp(-k_{-a}t)  \\ I(t) &=& I(0) exp (-k_{-i}t) +k_{i}bt/k_{-i} -\left[k_{i}b/(k_{-i})^{2}\right] (1-exp(-k_{-i}t)  \end{array} $$

but the key point is that the dominant behaviour of A is given by *A*∼*k*_*a*_*bt*/*k*_−*a*_ while that of I is given by *I*∼*k*_*i*_*bt*/*k*_−*i*_. Now examining the *R*^∗^ equation shows a linearly increasing forward and backward pathway which results in a quasi-steady state for the response *R*^∗^∼(*A*/*I*)/(*k*_*r*_/*k*_*f*_+*A*/*I*). The key point here is that even though A and I are increasingly asymptotically linearly in time their ratio *A*/*I* approaches a constant *k*_*a*_*k*_−*i*_/*k*_*i*_*k*_−*a*_ which is exactly the level in a constant stimulus (and it is independent of the stimulus). This implies that the response reaches a steady state corresponding to exact adaptation. This was noted in [52].

A very similar insight follows in the case of a quadratic ramp. There, by a very similar approach *A*∼*k*_*a*_*bt*^2^/*k*_−*a*_ and *I*∼*k*_*i*_*bt*^2^/*k*_−*i*_. For exactly the same reasons the response reaches a steady state. Asymptotically *A*/*I* reaches a steady level even though A and I are increasing, and the level it attains is exactly the value pre-stimulus: *k*_*a*_*k*_−*i*_/*k*_*i*_*k*_−*a*_. Thus exact adaptation is obtained in a quadratic ramp.

Now we consider an exponential stimulus *S*=*S*_0_*exp*(*λt*). The variation of A and I are given by

$$\begin{array}{@{}rcl@{}} A(t)&=& A(0)exp(-k_{-a}t) +(k_{a}S_{0}/(k_{-a}+ \lambda)[exp(\lambda t) -exp (-k_{-a}t)]  \\ I(t)&=& I(0)exp(-k_{-i}t) +(k_{i}S_{0}/(k_{-i}+ \lambda)[exp(\lambda t) -exp (-k_{-i}t)]  \end{array} $$

Both A and I are dominated by the increasing exponential terms *A*∼(*k*_*a*_*S*_0_/(*k*_−*a*_+*λ*)*exp*(*λt*) and *I*∼(*k*_*i*_*S*_0_/(*k*_−*i*_+*λ*)*exp*(*λt*). For the exact same reason as before, A/I reaches a steady value and the response reaches a steady state. Here however *A*/*I* asymptotes to a value *k*_*a*_(*k*_−*i*_+*λ*)/*k*_*i*_(*k*_−*a*_+*λ*) which is not the prestimulus level. In fact the higher the *λ* the greater the deviation from the exact adaptation.

**Adaptation in the mean to temporally periodic and static spatial stimuli.** In the text, we studied circuits which maintained their mean value in a periodic stimulus and also those which maintained the mean value in a static gradient. Inflow-outflow circuits (for instance two node motifs) could exhibit both, and a common structure allowed for this

$$\begin{array}{@{}rcl@{}} d/dt(A+B)&=& k_{0} -k_{2}B + k_{d} \frac{\partial^{2} A}{\partial \theta^{2}}  \end{array} $$

In a purely temporally periodic signal, the spatial term (diffusion) is zero, and the temporal term is zero when averaged. Exactly the reverse happens in a static spatial gradient, with the net effect that the averages are maintained, and this is true for other such circuits with similar structure.At any rate the presence of a control structure with constant coefficients is responsible.

The transcritical model does result in maintenance of mean value in a temporally periodic stimulus because the control structure is present as *dC*/*dt*=*C*(*k*_2_*B*−*k*_−2_): since this equation is separable, and so corresponds to a system with an integral control structure with constant coefficients. We have already seen that if A is diffusible, the spatial average of B will not be maintained: the main point is that in the absence of C diffusion, either *k*_2_*B*=*k*_−2_ (implying no gradient response) or *C*=0 (implying no maintenance of mean value of B: the latter happens when A is diffusible. The only remaining aspect to consider is what if C is diffusible? Rewriting the equation (assuming C is nonzero) and integrating over the domain using integration by parts

$$\begin{array}{@{}rcl@{}} k_{d} \frac{\partial^{2} C}{\partial \theta^{2}} [1/C] + k_{2}B-k_{-2}&=&0  \\ (1/L) \int_{0}^{L}(k_{2} B-k_{-2}) d \theta&=& (k_{d}/L) \int_{0}^{L} \left[-(dC/d\theta)^{2} \right]  \end{array} $$

This shows that the spatial average of B cannot be maintained, unless C is constant. One the other hand if C is constant, B cannot exhibit a gradient behaviour. This shows how fundamental constraints exist in this model, which also brings to the fore the difference between time and space, and the fact that space is associated with a second derivative which is ultimately what results in the deviation term on the RHS.

## Additional file

Additional file 1 Supplementary Material. (PDF 176 kb)
