# Supplementary material for: Adaptive information processing of network modules to dynamic and spatial stimuli
Source: BMC Syst Biol. 2019 Mar 14;13:32. doi: 10.1186/s12918-019-0703-1 (PMC6417070; doi:10.1186/s12918-019-0703-1)
Supplement: Supplementary file 1 — Supplementary Material. (PDF 176 kb) [file 12918_2019_703_MOESM1_ESM.pdf]

Adaptive information processing of network modules in response to dynamic and spatial stimuli: Supplementary Material

J. Krishnan [1,2] and Ioannis Floros [1]

[1] Department of Chemical Engineering, Centre for Process Systems Engineering and
[2] Institute for Systems and Synthetic Biology, Imperial College London, South Kensington Campus, London SW7 2AZ, UK.
j.krishnan@imperial.ac.uk. Ph: 44-20-7594-6633; Fax: 44-20-7594-6606.

Keywords: adaptation, dynamic stimuli, ramp, periodic response,

1 Organization of Supplementary material

The main text focussed on the response of adaptive circuits to a variety of time varying stimuli. The paper examined a range of models drawn from the literature in this regard. For the purposes of illustrating essential insights in the main text, a selection of circuits were presented to illustrate the main points. These results were backed up by analytical work in the Appendix. The Supplementary Material presents additional material supplementing the main text. This is organized as follows. In the next section we present model equations for the suite of models we have studied in the main text. In the following section we present additional plots for completeness, to complement those in the main text. We also round out our discussion in the main text with additional analysis.

We then present model equations for additional models we have studied. Finally we present the parameter values for the models used.

2 Models

This section is a summary of the primary models used in the main text including a short description of their characteristics and their mathematical equations. It consists of models which the paper has placed particular focus on. Other models that have been discussed less extensively have been placed in the secondary model section, which follows this section. The notation we adopt is for the input or signal to be represented by S and the nodes or species of the system by A, B, C and so on. Parameter notation is kept consistent with the relevant source to avoid confusion. The order in which the models are introduced is as follows DR08.M1, DR08.M1*, DR08.M1**, DR08.M31, DR08.M33, DR08.M34, DR12.M1, DR12.M4, KR09, KR11, C09.M2, KI14, MA09.FB and finally TC. The way these models are referred to provides an indication of the source as well as the particular model in that source. We point out at the outset, that the models as laid out here correspond to how they have been simulated. Other variations, including changes in where the signals act, or variations in the degree of reversibility in the network, have been considered and analyzed in the main text, with relevant models.

DR08.M1

A two-node open system. The reaction dynamics are represented by Mass Action kinetics. There is inflow into A and poentially B, while B also degrades (outflow). The signal mediates the conversion of A to B and is introduced in the place of the parameter k_2 in both A and B (in first-order reactions) and the output

considered is B. Note that a multiplicative rate constant (mediating the A to B conversion) can be absorbed in the signal. This is used in all the models below for the purpose of simulations.

$$\begin{aligned} \dot{A} &= k_{f1} - S \cdot A + k_{b2} \cdot B - k_{b1} \cdot A \\ \dot{B} &= S \cdot A - k_{f3} \cdot B - k_{b2} \cdot B + k_{b3} \end{aligned} \tag{1}$$

DR08.M1*

A two-node open system. The reaction dynamics are represented by Mass Action kinetics. There are no reversible and 3 irreversible reactions, of which 1 is zero order. The signal mediates the conversion of A to B and is introduced in the place of the parameter k_2 in both A and B (in a first-order reaction) and the output considered is B. This model can be seen as a special case of the earlier one.

$$\begin{aligned} \dot{A} &= k_1 - S \cdot A \\ \dot{B} &= S \cdot A - k_3 \cdot B \end{aligned} \tag{2}$$

DR08.M1**

A four-node open system with multiple negative feedback reactions. This is a slightly modified version of a model discussed in the literature, where certain interactions have been deactivated. There are no reversible and 6 irreversible reactions. The signal is introduced in the place of the parameter k_2 in both A and B (in a first-order reaction) and the output considered is B. The output adapts exactly in a step stimulus, as can be seen from a simple analysis.

$$\begin{aligned} \dot{A} &= \frac{k_1}{K_{11} + C} - S \cdot A \\ \dot{B} &= S \cdot A - k_3 \cdot B \\ \dot{C} &= k_4 - k_5 \cdot C \\ \dot{D} &= k_5 \cdot C - \frac{k_6 \cdot D}{K_{12} + A} \end{aligned} \tag{3}$$

DR08.M31

A three-node open, cyclic system. The reaction dynamics are represented by Mass Action kinetics. This is a cyclic motif involving three species with reversible reactions between the three species. In addition, thre is one inflow (to A) and two outflow (to B and C) reactions The signal is usually introduced in the place of the parameter k_{b2} or k_{f2} in both A and B (i.e. mediating the conversion between A and B in either direction) and the output considered is B. The action of signal at other locations has also been studied.

$$\begin{aligned} \dot{A} &= k_{f1} - (k_{f2} + k_{b6}) \cdot A + k_{f6} \cdot C + S \cdot B \\ \dot{B} &= k_{f2} \cdot A - (k_{f3} + k_{f4} + S) \cdot B + k_{b4} \cdot C \\ \dot{C} &= -(k_{f5} + k_{f6} + k_{b4}) \cdot C + k_{f4} \cdot B + k_{b6} \cdot A \end{aligned} \tag{4}$$

DR08.M32

This model is similar to the DR08.M31 system with the only difference being that the transition between B and C as well as that between C and A are irreversible. The signal is introduced in the place of the parameter k_{b2} or k_{f2} in both A and B (i.e. mediating the conversions between A and B) and the output considered is B. Other locations for the action of the signal have also been analyzed in the main text (see Appendix).

$$\begin{aligned}\dot{A} &= k_{f1} - (k_{f2} + k_{b6}) \cdot A + k_{f6} \cdot C + S \cdot B \\ \dot{B} &= k_{f2} \cdot A - (k_{f3} + k_{f4} + S) \cdot B \\ \dot{C} &= -(k_{f5} + k_{f6}) \cdot C + k_{f4} \cdot B \end{aligned} \tag{5}$$

DR08.M33

This model again is similar to the DR08.M31 system. The only difference here is that there are two inflow variables (A,C) and one outflow (B). The qualitative behaviour is determined by the fact that there is only one outflow. The signal is introduced in the place of the parameter k_{b2} or k_{f2} (i.e. mediating the conversions between A and B) in both A and B (in a first-order reaction) and the output considered is B. The action of the signal at other locations has also been studied (see analysis and discussion in the main text and Appendix).

$$\begin{aligned}\dot{A} &= k_{f1} - (k_{f2} + k_{b6}) \cdot A + k_{f6} \cdot C + S \cdot B \\ \dot{B} &= k_{f2} \cdot A - (k_{f3} + k_{f4} + S) \cdot B + k_{b4} \cdot C \\ \dot{C} &= k_{f5} - (k_{b4} + k_{f6}) \cdot C + k_{f4} \cdot B + k_{b6} \cdot A \end{aligned} \tag{6}$$

DR08.M34

This model again is similar to the DR08.M33 system. The only difference is that the transitions between B and C as well as C and A are irreversible. The signal is introduced in the place of the parameter k_{b2} or k_{f2} in both A and B (i.e. mediating the conversions between A and B) and the output considered is B.The action of the signal at other locations has also been studied (see analysis and discussion in the main text and Appendix).

$$\begin{aligned}\dot{A} &= k_{f1} - (k_{f2} + k_{b6}) \cdot A + k_{f6} \cdot C + S \cdot B \\ \dot{B} &= k_{f2} \cdot A - (k_{f3} + k_{f4} + S) \cdot B \\ \dot{C} &= k_{f5} - k_{f6} \cdot C + k_{f4} \cdot B \end{aligned} \tag{7}$$

DR08.M4

A closed system model, variant of a core element of the bacterial chemotaxis model introduced by Barkai-Leibler . In this case, however, there are 1 reversible and 2 irreversible reactions, of which 1 is zero order. Note that this model depicts a 3 species system, where the third species C is coupled to the other two (the system is closed) through conservation. The signal is introduced in the place of the parameter k_{b3} or k_{f3} in both A and B (in the reactions mediating the interconversion between A and B) and the output considered

is A.

$$\dot{A} = -(k_{f1} \cdot ChB + k_{b3}) \cdot A + k_{f3} \cdot B \cdot S$$
$$\dot{B} = k_{b3} \cdot A + k_{f2} \cdot ChR - k_{f3} \cdot B \cdot S \tag{8}$$

We note that the third node C could be explicitly incorporated, and we have studied this variant of the model as well. In general the same essential conclusions hold good. We note that when a ramp is applied to the variable A, as discussed, the model does not adapt: in fact the concentration of A approaches 0 and then so does C. In this case one must be careful and employ Michaelis-Menten equations, for the conversion of C to B (zeroth order approximation may not hold good). The same overall conclusion holds good.

DR12.M1

A two-node open system based on negative feedback and inflow control mechanisms. There are no reversible and 4 irreversible reactions, of which 2 are zero order. The signal is introduced in the place of the parameter k_{in} in A (in a zero-order reaction) and the output considered is A.

$$\dot{A} = S - k_{out} \cdot A + \frac{V_{max}^{B_{in}} \cdot A_{ext}}{K_M^{B_{in}} + A_{ext}} \cdot B$$
$$\dot{B} = k_s^B - \frac{V_{max}^{B_{set}} \cdot B}{K_M^{B_{set}} + B} \cdot A \tag{9}$$

DR12.M4

A two-node open system based on negative feedback and inflow control mechanisms. There are no reversible and 4 irreversible reactions, of which 2 are zero order. The signal is introduced in the place of the parameter k_{in} in A (in a zero-order reaction) and the output considered is A.

$$\dot{A} = S - k_{out} \cdot A + \frac{V_{max}^{B_{in}} \cdot A_{ext}}{K_M^{B_{in}} + A_{ext}} \cdot \frac{K_I^B}{K_I^B + B}$$
$$\dot{B} = k_s^B - \frac{V_{max}^{B_{set}} \cdot B}{K_M^{B_{set}} + B} \cdot \frac{K_I^A}{K_I^A + A} \tag{10}$$

This model exhibits close to exact adaptation in a step under conditions where $K_M^{B_{set}}, K_M^{B_{in}}$ are very small. This also corresponds to the parameter choice in the original model which has been used here in the simulations. Under these conditions, it is easy to see that the RHS of the equation for B is essentially independent of B (the Michaelis-Menten term reduces to a zeroth order kinetic term). In such a situation, at steady state, the steady state concentration of A is determined from this equation independent of the signal, explaining this behaviour. The steady state value of B does depend on the signal.

KR09

A three-node closed system based on incoherent feed forward characteristics. The reaction dynamics are represented by Mass Action kinetics. The signal is introduced in both A and B (in zero-order reactions) and

the output considered is R^*.

$$\begin{aligned}\dot{A} &= k_{fa} \cdot S - k_{ba} \cdot A \\ \dot{I} &= k_{fi} \cdot S - k_{bi} \cdot I \\ \dot{R^*} &= k_f \cdot A \cdot (1 - R^*) - k_r \cdot I \cdot R^* \end{aligned} \tag{11}$$

KR11

This model is similar to the previous model but with the added factor of saturation. The signal is introduced in both A and B (in first-order reactions) and the output considered is R^*.

$$\begin{aligned}\dot{A} &= k_{fa} \cdot S \cdot (A_{tot} - A) - k_{ba} \cdot A \\ \dot{I} &= k_{fi} \cdot S \cdot (I_{tot} - I) - k_{bi} \cdot I \\ \dot{R^*} &= k_f \cdot A \cdot (1 - R^*) - k_r \cdot I \cdot R^* \end{aligned} \tag{12}$$

CO09.M2

A three-node closed system with the characteristic of incoherent feed forward introduced by threshold terms. Additionally, 2 threshold terms exist: $H(th_2 - B)$ and $H(A - th_1)$. The signal is introduced in both A and B and the output considered is C. Note that this circuit implements an incoherent feedforward logic, but the threshold terms for A and B are a key ingradient.As a consequence if either a (sustained) signal is too low or too high, the output at steady state approaches zero.

$$\begin{aligned}\dot{A} &= k_f \cdot S \cdot (A_{tot} - A) - k_b \cdot A \\ \dot{B} &= k_f \cdot S \cdot (B_{tot} - B) - k_b \cdot B \\ \dot{C} &= b_1 \cdot H(A - th_1) \cdot H(th_2 - B) - a_1 \cdot C \end{aligned} \tag{13}$$

KI14

A three-node closed system based on incoherent feed forward characteristics. Additionally, the system exhibits approximately a parameter dependent fold change adaptation. This happens when p_2 is high, which we will assume and is reflected in the parameter values used. The signal is introduced in both A and B (in zero-order reactions) and the output considered is B.

$$\begin{aligned}\dot{A} &= S - A - p_1 \cdot (A \cdot B - C) \\ \dot{B} &= p_2 \cdot (S - A \cdot B) \\ \dot{C} &= p_3 \cdot (A \cdot B - C) \end{aligned} \tag{14}$$

MA09.FB

A three-node open system with negative feedback. The reaction dynamics are represented by Michaelis-Menten kinetics. The signal is introduced in A (in a first-order reaction) and the output considered is C.

$$\begin{aligned}\dot{A} &= S \cdot k_{IA} \cdot \frac{1-A}{1-A+K_{IA}} - F_A \cdot k'_{F_AA} \cdot \frac{A}{A+K'_{F_AA}} \\ \dot{B} &= C \cdot k_{CB} \cdot \frac{1-B}{1-B+K_{CB}} - F_B \cdot k'_{F_BB} \cdot \frac{B}{B+K'_{F_BB}} \\ \dot{C} &= A \cdot k_{AC} \cdot \frac{1-C}{1-C+K_{AC}} - B \cdot k'_{BC} \cdot \frac{C}{C+K'_{BC}} \end{aligned} \tag{15}$$

TC

A three-node closed system with the characteristic of transcritical bifurcation. The reaction dynamics are represented by Mass Action kinetics. Additionally, the third node, C, has the characteristic of autocatalysis. The signal is introduced in the place of the parameter k_{f1} in both A and$B$ (in first-order reactions) and the output considered is B.

$$\begin{aligned}\dot{A} &= k_{b1} \cdot B - S \cdot A \\ \dot{B} &= S \cdot A + k_{b2} \cdot C - (k_{b1} + k_{f2} \cdot C) \cdot B \\ \dot{C} &= k_{f2} \cdot B \cdot C - k_{b2} \cdot C \end{aligned} \tag{16}$$

3 Additional figures

The figures in the main text depicted the response of a selection of adaptive circuits, to concisely illustrate some key underlying points This section comprises of a section of additional results presented in a series of additional plots to round out the discussion in the main text.

In the main text, we presented a schematic depiction of the main models used in the text. In figure S1 we provide a schematic representation of models that were not presented (in detail) in the main figure 1. In particular we show a range of motifs with inflow and outflow (both two node motifs and three node motifs (panels A to G.)

In figure S2 extra model responses are presented for ramp (and step) signals. This figure is primarily related to figure 2 in the main paper. The response of two adaptive circuits (which exhibit contrasting responses in ramps in Fig. 2) to step stimuli is first presented. The step input response is shown here to confirm that the models do indeed adapt exactly to a step input. This consolidates the point made in the main text of how ramps can discriminate between fine differences in circuits. Fig. 2 illustrated a pair of circuits which exhibited different behaviour in a ramp. An additional comparison is given between two feedforward circuits KR11 and KR09, to show the effect on adaptation of having saturation. These two models are incoherent feedforward circuits which differ only in whether or not they have saturation. The effect of saturation in this context is clearly seen: in the absence of saturation exact adaptation ensues, while this is not the case with saturation. Finally, the effect of signal location in a motif was discussed in the main text showing how ramp stimuli associated with certain reactions may not lead to adaptive responses, while others do. An additional example of this kind is shown here, revealing how a ramp can result in an

increasing non-adaptive response, when applied to certain steps (the case shown in the main text is one of a decreasing non-adaptive response).

One of the circuits studied in the main text is a feedback motif presented by Ma and co-workers (MA09.FB). In figure S3 the effect on adaptation is studied in models for changes in feedback parameter, This figure is related to figure 4 in the main paper. In panels A and B, where small KAC values of 0.001 and 0.1 respectively have been used, it is observed that as the feedback coefficient is increased the adaptive steady state error decreases. Conversely, in panel C, where a KAC value of 10 has been used, the adaptive steady state error increases. The remaining part of this figure focusses on different 3 node motifs with inflow and outflow, focussing on ramps applied to two opposing reactions. We see responses ranging from exact adaptation in both locations in a 3 node motif (this happens in a completely reversible 3 node motif, where the only outflow node is the adapting variable), no adaptation in either location (the three node complete reversible motif with two outflow variables: incidentally this circuit does not exhibit exact adaptation in a step), and other cases where one location is associated with exact adaptation and the other is not (this complements what has been demonstrated in the main text for a particular case). We see how the network structure, location of the signal and the identities of outflow variables play crucial roles in this regard.

In figure S4 the effect of adaptation is studied with respect to a linear ramp, ramp capping and changes in the magnitude of the gradient of the signal. This figure is related to figure 5 in the main paper. From panel A, it is observed, similar to the main paper, that the response to a linear ramp and capped ramp is identical. Naturally this depends on the level of capping of the ramp, but indicates that adaptation in a ramp can occur independent of any capping. Panels B,C study a feedforward motif model without and with saturation, for ramps of different gradient strength. The model without saturation exhibits exact adaptation, though the transient profiles depend on the gradient strength. The model with saturation does not adapt, and the response also depends on the gradient strength (with some qualitative differences between profiles). Panels D and E study a model which exhibits fold adaptation, showing that different gradients elicit different transient responses (note that changing the basal level of input and gradient strength in proportion will keep the transient response unchanged in this model). Finally, the model considered in panel A is simulated by changing the basal level and gradient strength in proportion, showing that the transient response does change. This is typical of most circuits.

In figure S5 extra model responses to a periodic signal with changes to basal value are depicted. This figure is related to figure 6 in the main paper. Again similar observations are made here. It is observed, in panels A, B and F, that for some models the mean of oscillations is not maintained whereas in panels C, D, E and G other models are able to maintain or closely maintain the mean. Panel A depicts a feedforward model of inexact adaptation (over adaptation): we see the response being one of decreasing amplitude, but whose mean value increased and decreased. Panel B depicted a feedback motif, whose mean value increased. Panel F depicts a 3 node motif which is completely reversible. This is notable since, equivalent motifs which were partly reversible and partly irreversible, actually maintained the mean value of the output, as discussed in the main text. Regarding the models which maintain a mean value, we have a transcritical circuit (analyzed in the main text), other open system circuits (panel C and G), and E is another feedforward motif (where the mean is approximately maintained). This can be understood in terms of the analytical discussion in the main text.

In figure S6 extra model responses to a periodic signal with changes to amplitude value and changes both of amplitude and basal values at a fixed ratio are depicted. This figure is related to figure 6 and in the main paper (also see Fig. S7 below). In panels A-C the responses to changing signal amplitude are as expected, reflecting the ones seen in the main paper. While increasing amplitude leads to an increased amplitude in response, we also see how characteristics such as saturation play a role: panel A depicts an overadaptive feedforward motif model with saturation, showing a pronounced asymmetry in oscillations emerging with increased amplitude. Panel C shows a transcritical circuit whose mean value is maintained. The effect of varying mean value and amplitude together (over the range considered) shows that in the over adaptive motif

model, the amplitude decreased as did the mean value, in the feedback motif, the amplitude decreased while the mean value increased and in the transcritical circuit, the amplitude decreased while the mean value was maintained.

We also studied the effect of variation of both basal level and amplitude keeping their ratio fixed (Fig. S7). We note two basic points here. The first being that the CO09.M2 model shows a transition from no oscillations to oscillations, whose average and amplitude change, back to no oscillations. This reinforces the point that oscillatory signals can reveal distinct features of some circuits, along with the underlying structure. Theother point is the fact that one of models KI14 shows no change in the response, and this factor can be traced to the fact that this model exhibits fold-adaptation. This can be understood analytically.

Figure S8 deals with spatiotemporal inputs. Additional model responses to spatially graded ramps and periodic signals in spatial-temporal domain are illustrated. This figure is related to figure 8 in the main paper. Panel A demonstrates the response of an incoherent feedforward motif with diffusible inhibitor(the so-called LEGI module). We see that in a spatially graded ramp, we do see a graded response which reaches a steady state (the steady state profile is shown). In panel B, we consider a transcritical circuit with the autocatalytic species not diffusing. Here the mean value of the output is maintained, but the output is response to a travelling wave input may not be a travelling wave. However when a small diffusivity is introduced, the output becomes a travelling wave, but the output does not exactly maintain the mean value.

4 Additional Comments on models.

Our analysis in the main text and the supplementary material has focussed on a range of models, which have been studied using simulations and analytical work. We present some additional comments on these models, to synthesize the insights which emerge.

Feedforward motifs. A number of feedforward motif models have been analyzed including KR09, KR11, CO09.M1, CO09.M2, MA09FF, KI14. It is clear that within a feedforward characteristic, multiple variations are possible and this can affect the way information processing occurs in these models. With regard to ramp response, we have shown how Model KR09 adapts, essentially due to cancellation of the contributions of the feedforward legs of the circuit (even though they dont individually reach steady state). KR11, contains the effect of saturation, so in a ramp, adaptation can only be inexact. Taken together it shows that in model KR11, the only reason why adaptation is not exact is due to saturation.

If we look at the model CO09M1 (see equation below), we find that the adapting variable is faced with two contributions, one associated with an increase of signal, and one with a threshold function which switches off the effect through the other leg. Thus in a ramp the adapting variable B will approach zero, because of the presence of the Heaviside function which switches off, when the signal (and consequently A) become high. In contrast in the model CO09.M2, the two feedforward legs are both associated with Heaviside functions, but in different directions: one switches on when the variable crosses a threshold (the variable A) and the other one switches off (the variable B).So if the stimulus is below the threshold of A to start with, in a ramp, it will end up switching off the leg associated with the variable B, and this will lead to a zero output and consequently exact adaptation. MA09.FF is a 3 node feedforward motif, which adapts imperfectly, because of the presence of saturation. Finally the model KI14, also exhibits exact adaptation in a ramp. In this model, the situation in reminiscent of the KR09 model, where the two legs (in this case A and C) do not reach a steady state but the output B reaches a steady state due to the cancellation of the two effects. We briefly demonstrate this behaviour, which is seen in simulations.

Changing variables to $A_1 = A/S, B_1 = B, C_1 = C/S$ we find that the equations for the model can be

written as

$$
\begin{aligned}
dA_1/dt &= 1 - A_1 - p_1(A_1B_1 - C_1) - (A_1/S)dS/dt \\
dB_1/dt &= p_2S(1 - A_1B_1) \\
dC_1/dt &= p_3(A_1B_1 - C_1) - (C_1/S)dS/dt
\end{aligned} \tag{17}
$$

For signals like linear ramps and quadratic ramps (but not exponential signals), the last term in the first and third equations can be neglected, relative to other terms of the same type in the equation and this results in the equation:

$$
\begin{aligned}
dA_1/dt &= 1 - A_1 - p_1(A_1B_1 - C_1) \\
dB_1/dt &= p_2S(1 - A_1B_1) \\
dC_1/dt &= p_3(A_1B_1 - C_1)
\end{aligned} \tag{18}
$$

Now here the signal appears only in the second equation and as a multiplicative term. Since simulations indicate that B (equal to B_1) reaches a steady state, we see that the only way that can happen is if A_1B_1 asymptotes to 1. Now the other two equations do not involve the signal, and so the other two variables will reach a steady state (in fact as long as B_1 asymptotes to a finite value). In particular, from the third equation we see that C_1 approaches 1, and from the first equation, so does A_1. This explains the observation that A and C are increasing linearly with time asymptotically with time, while B adapts due to the cancellation of the two effects.

We can explore this a little further. We note straightaway that A_1B_1 cannot asymptote to any other steady value (including zero): if it did, we find that this is the value C_1 would asymptote to, and from the first equation A_1 would approach 1, but that would be inconsistent with an increasing B. Furthermore, the dynamics of this model are constrained by the fact that no variable can go below zero, unless some other has already gone below zero, which means that no variable can go below zero. This and the above analysis constrains the asymptotic behaviour. Finally the fact that exact adaptation does not occur in an exponential signal can also be understood. In fact we see the extra terms in the first and third equations are not negligible. Now if B_1 reaches a steady state that would imply that (i)A_1B_1 would have to asymptote to 1, and (ii)C_1 would have to asymptote to a value different from 1 (dependent only on p_3). This would mean then that A_1 would asymptote to a value different from 1, and consequently B_1 would asymptote to a value different from 1, meaning that the adaptation is inexact.

Feedback motifs. We primarily analyzed two feedback motifs, one the model of MA09.FB. Here computational analysis shows that adaptation in a ramp is not exact (as may be expected). This analysis has been performed in the main text. Another feedback motif considered was a model mimicking E.coli chemotactic signalling: model DR08.M4 (see discussion of model). This is a closed system model, which contains one step acting via zeroth order reaction. This model has 3 species, a pair involving reversible reactions (associated with the variables A and B) and a third, which is linked to species A and B by irreversible reactions. The conversion of this species to B acts by a zeroth order reaction. Now in this case, a ramp signal associated with B to A conversion will lead to exact adaptation (essentially due to short circuiting of a step), but a ramp associated with A to B will not, for reasons we have seen in other inflow-outflow circuits. Thus exact adaptation is seen only in one direction. This shows how even within a particular characteristic (feedback), the exact way it is realized and the ingredients involved can play important roles. In fact this model shares the common characteristics of inflow-outflow models (apart from the fact that it is closed) and its underlying behaviour can be seen as a direct parallel of that in similar inflow-outflow models.

Inflow-Outflow circuits. We consider three node inflow outflow motifs in some detail. As presented earlier this includes cases of a single outflow (model DR08.M33, which is completely reversible, and model DR08.M34, which is partially reversible (B converts to C and C to A)) and two outflows (model DR08.M31,

which is completely reversible, and model DR08.M32, which like DR08.M34, is partially reversible). We contrast the behaviour of all these models. Firstly, in the case of a ramp, model DR08.M31 does not exhibit exact adaptation whatever reaction the signal is associated with. We note that in model DR08.M31, even in a step, exact adaptation does not result. We note in this two-outflow motif that the requirement that inflow matches outflow only puts a constraint on a combination of B and C, which by itself does not guarantee adaptation of B. If we move to model DR08.M32, we find that exact adaptation of B ensues if the signal is associated with the A to B conversion. The signal in the other locations affects the asymptotic concentration of C (or is associated with the degradation of B), and consequently prevents exact adaptation. Now if we turn to the the single outflow cases, we note that if the entire system is at a steady state then B should adapt (since inflow should match outflow). We find that a ramp signal associated with 4 of the 6 reactions will give exact adaptation: the two reactions associated with B conversion will not. This aspect is already understood from our study of two-node motifs. Finally we find that in model DR08.M34, again the two reactions associated with B conversion are those which will not give exact adaptation in a ramp. These aspects are also studied in detail in the Appendix in multiple ways.

Now in periodic stimuli, we find that in model DR08.M31, the mean of the output is not maintained irrespective of the location of the signal. In model DR08.M32, the mean is maintained when the signal is associated with A to B (or reverse) conversion only. This is understood by noting that the periodicity of the output only imposes that a combination of the time-average of B and that of C is fixed. Any signal which leaves the balance of the time averages of B and C untouched can maintain the mean of the output, and this happens only when the signal is associated with the A and B interconversion. For the case of the single outflow models, the time average of the output is maintained for all locations.

Taken together, this indicates that moving from one outflow to two outflow variables generally imposes more constraints for both adaptation in a ramp and maintenance of mean value in periodic stimuli, and further increased reversibility generally imposes additional constraints as well.

Spatial stimuli. In the text we asserted that in a transcritical model with species A diffusing would give a gradient response. In the notation of the analysis in the main text we have

$$
\begin{aligned}
dA/dt &= -k_1SA + k_2B + k_d\frac{\partial^2 A}{\partial\theta^2} \\
dB/dt &= k_1SA - k_2B - k_3BC + k_4C \\
dC/dt &= k_3BC - k_4C
\end{aligned}
\tag{19}
$$

We mentioned in the context of simulations that we obtain $C = 0$, whhich is what allows for a gradient behaviour of the output B. Here we show that a solution with non-zero C cannot be obtained. We assume that A is highly diffusible. Now if $C > 0$ then from the final equation $B = k_4/k_3$. From the first equation since A is highly diffusible it will attain, an essentially uniform steady state. Then from the second equation at steady state, we must have $k_1SA = k_2k_4/k_3$ which cannot be satisfied in general when S is spatially inhomoegenous. Thus the steady state obtained involves $C = 0$, which then automatically provides a gradient response for B: however, the spatial average of B is no longer maintained.

5 Model Equations - Secondary

The models that have been included here are DR08.M2, DR08.M32, DR08.M4, DR08.M6, DR08.M7, C09.M1, C09.M3, MA09.FF, DR12.M2, DR12.M3, DR12.M5, DR12.M6, DR12.M7 and finally DR12.M8.

DR08.M2

A multiple-node (i.e. N species) open system with consecutive linear reactions. The reaction dynamics are represented by Mass Action kinetics. There are no reversible and N+1 irreversible reactions, of which 1

is zero order. The signal is introduced in the place of the parameter k_2 in both A and B (in a first-order reaction) and the output considered is E the final node in this case (the model depicts a 5 stage reaction).

$$\begin{aligned}
\dot{A} &= k_1 - S \cdot A \\
\dot{B} &= S \cdot A - k_3 \cdot B \\
\dot{C} &= k_3 \cdot B - k_4 \cdot C \\
\dot{D} &= k_4 \cdot C - k_5 \cdot D \\
\dot{E} &= k_5 \cdot D - k_6 \cdot E
\end{aligned} \tag{20}$$

DR08.M6

A N-node open system with negative feedback (equations are shown for a 5 stage reaction). The reaction dynamics are represented by Mass Action kinetics. There are no reversible and N+1 irreversible (reactions, of which 1 is zero order. The signal is introduced in the place of the parameter k_2 in both A and B (in a first-order reaction) and the output considered is B.

$$\begin{aligned}
\dot{A} &= \frac{k_1}{K1 + E} - S \cdot A \\
\dot{B} &= S \cdot A - k_3 \cdot B \\
\dot{C} &= k_3 \cdot B - k_4 \cdot C \\
\dot{D} &= k_4 \cdot C - k_5 \cdot D \\
\dot{E} &= k_5 \cdot D - k_6 \cdot E
\end{aligned} \tag{21}$$

DR08.M7

A N-node open system where the reaction dynamics are represented by Mass Action kinetics (equations are shown for a 5 stage reaction). There are no reversible and N+1 irreversible reactions, of which 1 is zero order. The signal is introduced in the place of the parameter k_2 in both A and B (in a first-order reaction) and the output considered is B

$$\begin{aligned}
\dot{A} &= k_1 - S \cdot A \\
\dot{B} &= S \cdot A - k_3 \cdot B \\
\dot{C} &= k_3 \cdot B - k_4 \cdot C \\
\dot{D} &= k_4 \cdot C - k_5 \cdot D \cdot K_a \cdot B \\
\dot{E} &= k_5 \cdot D \cdot K_a \cdot B - k_6 \cdot E
\end{aligned} \tag{22}$$

DR12.M2

A two-node open system based on negative feedback and inflow control mechanisms. There are no reversible and 4 irreversible reactions, of which 1 is zero order. The signal is introduced in the place of the parameter k_{in} in A (in a zero-order reaction) and the output considered is A.

$$\dot{A} = S - k_{out} \cdot A + \frac{V_{max}^{B_{in}} \cdot A_{ext}}{K_M^{B_{in}} + A_{ext}} \cdot \frac{K_I^B}{K_I^B + B}$$
$$\dot{B} = k_s^B \cdot A - \frac{V_{max}^{B_{set}} \cdot B}{K_M^{B_{set}} + B} \tag{23}$$

DR12.M3

A two-node open system based on negative feedback and inflow control mechanisms. There are no reversible and 4 irreversible reactions, of which 1 is zero order. The signal is introduced in the place of the parameter k_{in} in A (in a zero-order reaction) and the output considered is A.

$$\dot{A} = S - k_{out} \cdot A + \frac{V_{max}^{B_{in}} \cdot A_{ext}}{K_M^{B_{in}} + A_{ext}} \cdot B$$
$$\dot{B} = k_s^B \cdot \frac{K_I^A}{K_I^A + A} - \frac{V_{max}^{B_{set}} \cdot B}{K_M^{B_{set}} + B} \tag{24}$$

DR12.M5

A two-node open system based on negative feedback and outflow control mechanisms. There are no reversible and 4 irreversible reactions, of which 1 is zero order. The signal is introduced in the place of the parameter k_{in} in A (in a zero-order reaction) and the output considered is A.

$$\dot{A} = S - k_{out} \cdot A - \frac{V_{max}^{B_{out}} \cdot A_{ext}}{K_M^{B_{out}} + A_{ext}} \cdot B$$
$$\dot{B} = k_s^B \cdot A - \frac{V_{max}^{B_{set}} \cdot B}{K_M^{B_{set}} + B} \tag{25}$$

DR12.M6

A two-node open system based on negative feedback and outflow control mechanisms. There are no reversible and 4 irreversible reactions, of which 2 are zero order. The signal is introduced in the place of the parameter k_{in} in A (in a zero-order reaction) and the output considered is A.

$$\dot{A} = S - k_{out} \cdot A - \frac{V_{max}^{B_{out}} \cdot A_{ext}}{K_M^{B_{out}} + A_{ext}} \cdot \frac{K_I^B}{K_I^B + B}$$
$$\dot{B} = k_s^B - \frac{V_{max}^{B_{set}} \cdot B}{K_M^{B_{set}} + B} \cdot A \tag{26}$$

DR12.M7

A two-node open system based on negative feedback and outflow control mechanisms. There are no reversible and 4 irreversible reactions, of which 2 are zero order. The signal is introduced in the place of the

parameter k_{in} in A (in a zero-order reaction) and the output considered is A.

$$\dot{A} = S - k_{out} \cdot A - \frac{V_{max}^{B_{out}} \cdot A_{ext}}{K_M^{B_{out}} + A_{ext}} \cdot B$$
$$\dot{B} = k_s^B - \frac{V_{max}^{B_{set}} \cdot B}{K_M^{B_{set}} + B} \cdot \frac{K_I^A}{K_I^A + A} \tag{27}$$

DR12.M8

A two-node open system based on negative feedback and outflow control mechanisms. There are no reversible and 4 irreversible reactions, of which 1 is zero order. The signal is introduced in the place of the parameter k_{in} in A (in a zero-order reaction) and the output considered is A.

$$\dot{A} = S - k_{out} \cdot A - \frac{V_{max}^{B_{out}} \cdot A_{ext}}{K_M^{B_{out}} + A_{ext}} \cdot \frac{K_I^B}{K_I^B + B}$$
$$\dot{B} = k_s^B \cdot \frac{K_I^A}{K_I^A + A} - \frac{V_{max}^{B_{set}} \cdot B}{K_M^{B_{set}} + B} \tag{28}$$

CO09.M1

A two-node closed system with the characteristic of incoherent feed forward introduced by a threshold term, $H(th - A)$. The signal is introduced in both A and B (in zero-order reactions) and the output considered is B.

$$\dot{A} = b_1 \cdot S - a_1 \cdot A$$
$$\dot{B} = b_1 \cdot S \cdot H(th - A) - a_1 \cdot B \tag{29}$$

CO09.M3

A six-node closed system with the characteristic of incoherent feed forward introduced by threshold terms. The reaction dynamics are represented by Mass Action kinetics. There are no reversible and 8 irreversible reactions. Additionally, 6 threshold terms exist: $H(A - th_1)$, $H(th_2 - B)$, $H(C - th_3)$, $H(C - th_4)$, $H(D - th_5)$ and $H(th_6 - E)$. The signal is introduced in both A and B (in zero-order reactions) and the output considered is F.

$$\dot{A} = b_1 \cdot S - a_1 \cdot A$$
$$\dot{B} = b_1 \cdot S - a_1 \cdot B$$
$$\dot{C} = b_1 \cdot H(A - th_1) \cdot H(th_2 - B) - a_1 \cdot C$$
$$\dot{D} = b_2 \cdot H(C - th_3) - a_2 \cdot D$$
$$\dot{E} = b_2 \cdot H(C - th_4) - a_2 \cdot E$$
$$\dot{F} = b_2 \cdot H(D - th_5) \cdot H(th_6 - E) - a_2 \cdot F \tag{30}$$

MA09.FF

A three-node open system with an incoherent feed-forward loop. The reaction dynamics are represented by Michaelis-Menten kinetics. The signal is introduced in the place of the parameter k_2 in A (in a first-order reaction) and the output considered is C.

$$
\begin{aligned}
\dot{A} &= S \cdot k_{IA} \cdot \frac{1-A}{1-A+K_{IA}} - F_A \cdot k'_{F_A A} \cdot \frac{A}{A+K'_{F_A A}} \\
\dot{B} &= A \cdot k_{AB} \cdot \frac{1-B}{1-B+K_{AB}} - F_B \cdot k'_{F_B B} \cdot \frac{B}{B+K'_{F_B B}} \\
\dot{C} &= A \cdot k_{AC} \cdot \frac{1-C}{1-C+K_{AC}} - C \cdot k'_{BC} \cdot \frac{C}{C+K'_{BC}}
\end{aligned} \tag{31}
$$

6 Model parameters - Primary

We begin by specifying the signal used. For a ramp signal, this is done by introducing a basal signal of magnitude 0.1 and then introducing a ramp at time $t = 50$. The slope of the ramp is 1 unless otherwise mentioned. Similarly when a quadratic or exponential ramp is imposed, this is done in an exactly analogous way (with multiplicative factor 1, unless otherwise mentioned). For periodic signals, we impose signals of the form $S = a + bsinwt$. When the variation of mean value of the stimulus is studied, the amplitude $b = 0.1$.When the amplitude is varied keeping the mean value fixed, the mean value is taken to be $a = 1$. When we vary a and b together in proportion, $b/a = 0.4$. Variations of parameters are indicated in the corresponding plots. For spatial signals imposed on a domain where x varies from 0 to 1,we employ signals of the form (i) $S = (a + bcos(2\pi x))t$, where $a = 2, b = 0.75$: this a spatial gradient combined with a temporal ramp (ii) $S = a + bcoswtcos(2\pi x)$ for the same values of a and b below, and $w = 0.1$ (iii)$S = a + bcos(2\pi x - dt)$ with $d = 0.01$. In the case of spatial signals, when a species is diffusible, its dimensionless diffusion coefficient is set to 1. In the case of the transcritical model we have studied the case where the autocatalytic variable is non-diffusible and also when it is moderately diffusible (dimensionless diffusion coefficient=0.1). In this model, A is assumed to be diffusible.

DR08.M1

Parameter	Value
k_{f1}	1
k_{f2}	S
k_{f3}	1
k_{b1}	0
k_{b2}	1
k_{b3}	1

DR08.M1*

Parameter	Value
k_1	1
k_2	S
k_3	0.5

DR08.M1**

Parameter	Value
k_1	1
k_2	S
k_3	1
k_4	1
k_5	1
k_6	1
k_{11}	0.5
k_{12}	0.35

DR08.M3

Parameter	Value
k_{f1}	1.1
k_{f2}	1.2
k_{f3}	1.3
k_{f4}	1.4
k_{f5}	1.5
k_{f6}	0.1
k_{b2}	1.7
k_{b4}	1.8
k_{b6}	1.9

DR12.M1

Parameter	Value
k_{in}	S
k_{out}	1
$V_{max}^{B_{in}}$	0.5
A_{ext}	2
$K_M^{B_{in}}$	0.0001
k_s^B	1
$V_{max}^{B_{set}}$	0.5
$K_M^{B_{set}}$	0.001

DR12.M4

Parameter	Value
k_{in}	S
k_{out}	2
$V_{max}^{B_{in}}$	10
A_{ext}	1
$K_M^{B_{in}}$	0.0001
K_I^A	0.64
K_I^B	0.01
k_s^B	0.5
$V_{max}^{B_{set}}$	1
$K_M^{B_{set}}$	0.001

KR09

Parameter	Value
k_{fa}	1.5
k_{ba}	2
k_{fi}	0.8
k_{bi}	1.3
k_f	1.1
k_r	1.7

KR11

Parameter	Value
k_{fa}	1.5
k_{ba}	2
k_{bi}	1.7
k_f	1.3
k_r	1.7
A_{tot}	20
B_{tot}	20

CO09.M2

Parameter	Value
k_f	4
k_b	2
b_1	10
a_1	0.01
A_{tot}	800
B_{tot}	800
th_1	500
th_2	500

KI14

Parameter	Value
p_1	0.01
p_2	200
p_3	0.01

MA09.FB

Parameter	Value
k_{IA}	1
k_{CB}	1
k_{AC}	10
K_{IA}	1
K_{CB}	0.01
K_{AC}	0
k'_{F_AA}	1
k'_{F_BB}	1
k'_{BC}	20
K'_{F_AA}	1
K'_{F_BB}	0.01
K'_{BC}	1
F_A	1
F_B	1

TC

Parameter	Value
k_{f1}	S
k_{f2}	1.4
k_{b1}	0.4
k_{b2}	0.2

7 Model Parameters-Secondary

DR08.M2

Parameter	Value
k_1	1
k_2	S
k_3	1
k_4	1
k_5	1
k_6	1

DR08.M4

Parameter	Value
k_{f1}	0.05
k_{f2}	0.5
k_{f3}	1
k_{b3}	1
ChB	3
ChR	3

DR08.M6

Parameter	Value
k_1	1
k_2	S
k_3	1
k_4	1
k_5	1
k_6	1
K1	1

DR08.M7

Parameter	Value
k_1	1
k_2	S
k_3	1
k_4	1
k_5	1
k_6	1
Ka	1

DR12.M2

Parameter	Value
k_{in}	S
k_{out}	1
$V_{max}^{B_{in}}$	10
A_{ext}	2
$K_M^{B_{in}}$	0.0001
K_I^B	0.01
k_s^B	0.5
$V_{max}^{B_{set}}$	1
$K_M^{B_{set}}$	0.001

DR12.M3

Parameter	Value
k_{in}	S
k_{out}	15
$V_{max}^{B_{in}}$	10
A_{ext}	1
$K_M^{B_{in}}$	0.0001
K_I^A	0.64
k_s^B	0.5
$V_{max}^{B_{set}}$	0.5
$K_M^{B_{set}}$	0.001

DR12.M5

Parameter	Value
k_{in}	S
k_{out}	5
$V_{max}^{B_{out}}$	0.5
$K_M^{B_{out}}$	0.0001
k_s^B	0.5
$V_{max}^{B_{set}}$	1
$K_M^{B_{set}}$	0.001

DR12.M6

Parameter	Value
k_{in}	S
k_{out}	1
$V_{max}^{B_{out}}$	10
$K_M^{B_{out}}$	0.0001
K_I^B	0.01
k_s^B	0.5
$V_{max}^{B_{set}}$	0.5
$K_M^{B_{set}}$	0.001

DR12.M7

Parameter	Value
k_{in}	S
k_{out}	25
$V_{max}^{B_{out}}$	10
$K_M^{B_{out}}$	0.0001
K_I^A	0.64
k_s^B	1
$V_{max}^{B_{set}}$	1
$K_M^{B_{set}}$	0.001

DR12.M8

Parameter	Value
k_{in}	S
k_{out}	15
$V_{max}^{B_{out}}$	1
$K_M^{B_{out}}$	0.0001
K_I^A	0.64
K_I^B	0.01
k_s^B	0.5
$V_{max}^{B_{set}}$	1
$K_M^{B_{set}}$	0.001

CO09.M1

Parameter	Value
b_1	10
a_1	0.01
th	800

CO09.M3

Parameter	Value
a_1	0.01
b_1	10
a_2	0.006
b_2	6
th_1	500
th_2	800
th_3	50
th_4	50
th_5	500
th_6	800

MA09.FF

Parameter	Value
k_{IA}	1
k_{AB}	0.5
k_{AC}	1
K_{IA}	1
K_{AB}	0.001
K_{AC}	1
k'_{F_AA}	1
k'_{F_BB}	10
k'_{BC}	1
K'_{F_AA}	1
K'_{F_BB}	100
K'_{BC}	1
F_A	1
F_B	1

Fig.S1 Schematic of circuits Schematic representation of extra circuits used for the research conducted in this paper: A. DR08.M1*, B. DR08.M1**, C. DR08.M31, D. DR08.M32, E. DR08.M33, F. DR08.M34, G. DR12.M1, H. MA09.FF.

Fig.S2 Responses to step and ramp inputs Models response to a small step signal: A. DR12.M4 adapts exactly, B. DR08.M**mod adapts exactly. These two circuits exhibit different responses in a ramp as discussed in the text. C. KR11 adapts close to exactly, D. KR09 adapts exactly Models' responses to a linear ramp signal: E. KR11 adapts inexactly, F. KR09 adapts exactly. G. DR08.M34 does not adapt reaching a SS below the initial SS, if the siganl is associated with the B to A reaction. I,J. The response of the model KI14 to a ramp. The output adapts while the two feedforward legs do not reach a steady state.

Fig.S3 Response of a feedback motif model and effect of signal location Sensitivity of feedback coefficient and KAC parameter in MA09.FB on adaptation to step and linear ramp signals. A. Response to a step signal ($KAC = 0.001$) B. Response to a linear ramp signal ($KAC = 0.1$) C. Response to a linear ramp signal ($KAC = 10$) Plots depicting how the location at which the signal acts can affect adaptation to step and linear ramp signals. D. Response of DR08.M33 to a step signal: if signal in k_{f2} exact adaptation, if signal in k_{b2} exact adaptation. E. Response of DR08.M31 to a linear ramp signal: if signal in k_{f2} no adaptation, if signal in k_{b2} no adaptation. F. Response of DR08.M32 to a linear ramp signal: if signal in k_{f2} exact adaptation, if signal in k_{b2} no adaptation. G. Response of DR08.M34 to a linear ramp signal: if signal in k_{f2} exact adaptation, if signal in k_{b2} no adaptation.

Fig. S4 Response of models to ramps and effect of ramp characteristics. Comparison of model responses between a linear ramp signal, capping of ramp and basal-gradient parameter variations. A. DR08.M1* adapts exactly to a ramp capping signal. The transient response is also identical to the ramp signal without capping. B. KR09 adapts exactly to a range of gradient values of the linear ramp signal, though the transient response depends on the strength of the gradient. C. KR11 adapts inexactly to a range of gradient values of the linear ramp signal. D. KI14 adapts exactly to a range of gradient values of the linear ramp signal, for a basal value of 0.1. E. KI14 adapts exactly to a range of gradient values of the linear ramp signal, for a basal value of 0.5. F. DR08.M1* adapts exactly to a range

of gradient and basal values varied at a fixed ratio ($grad/bsl = 0.4, grad = 0.04, 0.16, 2.56$). The transient responses in (D-F) vary with the change in signal characteristics.

Fig.S5 Response to a periodic stimulus: effect of mean value of input Effect of the basal level (mean value) of a periodic signal. Typical behaviour is one where amplitude of oscillations decreases with increase in basal value of signal. A. KR11 with chosen parameters that give over-adaptation. Typical behaviour with the mean of oscillations not maintained. B. MA09.FB exhibits typical behaviour with the mean of oscillations not maintained. C. FE16.M5 exhibits typical behaviour with the mean of oscillations maintained. D. TC exhibits typical behaviour with the mean of oscillations maintained. E. KI14 exhibits typical behaviour with the mean of oscillations close to maintained. F. DR08.M31 exhibits atypical behaviour, the amplitude of oscillations remains roughly constant, with the mean of oscillations not maintained. G. DR08.M33 exhibits typical behaviour with the mean of oscillations maintained.

Fig. S6 Response to periodic stimulus: variation of amplitude. Effect of varying the amplitude of a periodic signal. Typical behaviour is one where oscillations amplitude increase with increase in amplitude value of signal. A. KR11 with chosen parameters that give over-adaptation. Typical behaviour with the mean of oscillations not maintained. A pronounced asymmetry is seen. B. MA09.FB exhibits typical behaviour with the mean of oscillations not maintained. C. TC exhibits typical behaviour with the mean of oscillations maintained. Plots illustrating the effect on models output of varying the basal level and amplitude (together, in a fixed ratio of amplitude of basal of 0.4) of a periodic signal. D. KR11 with chosen parameters that give over-adaptation. Typical behaviour with the mean of oscillations not maintained. E. MA09.FB exhibits typical behaviour with the mean of oscillations not maintained. F. TC exhibits typical behaviour with the mean of oscillations maintained.

Fig. S7 Response to periodic stimulus: variation of amplitude and mean value in a fixed ratio. The effect of simultaneously varying the mean value and amplitude in a fixed ratio is examined, for the models discussed in the main text. Changing the mean value and amplitude together, involves an interplay between the contributions of each. A range of responses is seen: A. DR08.M1 shows a decrease in amplitude of oscillations with mean of oscillations being maintained. B. KR09 shows an increase in amplitude but a decrease in mean value of oscillations C. KR11 shows a decrease in amplitude but an increase in mean value D. CO09.M2 shows a reduction in amplitude while the mean of oscillations reaches a maximum and then decrease. E. KI14 maintains a fixed oscillatory output.

Fig. S8 Response to spatiotemporal stimuli. Responses of models to spatiotemporal stimuli. A. KR09 exhibits gradient sensing to a spatial ramp signal, when the inhibitor is diffusible. B. TC response becomes more like a travelling wave when the autocatalytic variable C is weakly diffusing (in addition to A which is diffusible).
